# Megakaryocytic IGF1 coordinates activation and ferroptosis to safeguard hematopoietic stem cell regeneration after radiation injury

**DOI:** 10.1186/s12964-024-01651-5

**Published:** 2024-05-27

**Authors:** Weinian Liao, Xinliang Chen, Shuzhen Zhang, Jun Chen, Chaonan Liu, Kuan Yu, Yimin Zhang, Mo Chen, Fang Chen, Mingqiang Shen, Binghui Lu, Songling Han, Song Wang, Junping Wang, Changhong Du

**Affiliations:** State Key Laboratory of Trauma and Chemical Poisoning, Institute of Combined Injury, Chongqing Engineering Research Center for Nanomedicine, College of Preventive Medicine, Army Medical University, Chongqing, 400038 China

**Keywords:** Hematopoietic stem cell, Megakaryocyte, IGF1, Ferroptosis, Ionizing radiation, Myelosuppression

## Abstract

**Background:**

Hematopoietic stem cell (HSC) regeneration underlies hematopoietic recovery from myelosuppression, which is a life-threatening side effect of cytotoxicity. HSC niche is profoundly disrupted after myelosuppressive injury, while if and how the niche is reshaped and regulates HSC regeneration are poorly understood.

**Methods:**

A mouse model of radiation injury-induced myelosuppression was built by exposing mice to a sublethal dose of ionizing radiation. The dynamic changes in the number, distribution and functionality of HSCs and megakaryocytes were determined by flow cytometry, immunofluorescence, colony assay and bone marrow transplantation, in combination with transcriptomic analysis. The communication between HSCs and megakaryocytes was determined using a coculture system and adoptive transfer. The signaling mechanism was investigated both in vivo and in vitro, and was consolidated using megakaryocyte-specific knockout mice and transgenic mice.

**Results:**

Megakaryocytes become a predominant component of HSC niche and localize closer to HSCs after radiation injury. Meanwhile, transient insulin-like growth factor 1 (IGF1) hypersecretion is predominantly provoked in megakaryocytes after radiation injury, whereas HSCs regenerate paralleling megakaryocytic IGF1 hypersecretion. Mechanistically, HSCs are particularly susceptible to megakaryocytic IGF1 hypersecretion, and mTOR downstream of IGF1 signaling not only promotes activation including proliferation and mitochondrial oxidative metabolism of HSCs, but also inhibits ferritinophagy to restrict HSC ferroptosis. Consequently, the delicate coordination between proliferation, mitochondrial oxidative metabolism and ferroptosis ensures functional HSC expansion after radiation injury. Importantly, punctual IGF1 administration simultaneously promotes HSC regeneration and hematopoietic recovery after radiation injury, representing a superior therapeutic approach for myelosuppression.

**Conclusions:**

Our study identifies megakaryocytes as a last line of defense against myelosuppressive injury and megakaryocytic IGF1 as a novel niche signal safeguarding HSC regeneration.

**Supplementary Information:**

The online version contains supplementary material available at 10.1186/s12964-024-01651-5.

## Background

Bone marrow (BM) hematopoiesis carries out vital immune, oxygen transport, hemostasis functions, and therefore must be rigorously regulated. The rare hematopoietic stem cells (HSCs) residing in the BM drive hematopoietic replenishment at homeostasis and hematopoietic regeneration after injury [1]. Normally, the pool size of HSCs is stably maintained at steady state. In the face of myelosuppressive injury induced by cytotoxicity of physical, chemical or biological origins, such as that suffered by patients receiving cancer therapies or HSC transplantation, albeit the vast majority of BM cells (BMCs) including HSCs are ablated, very few HSCs survive. These HSCs then undergo self-renewal proliferation to regenerate themselves and to reconstruct the whole hematologic and immune systems [2]. Despite that targeting HSC regeneration has attracted substantial attention in the treatment of myelosuppression, the mechanisms that regulate HSC regeneration remain incompletely understood, with a lack of effective countermeasures against myelosuppression.

Radiation injury has long been appreciated as a paradigm for the investigation of myelosuppressive injury, as HSCs are extremely sensitive to ionizing radiation (IR) [3–5]. When exposed to IR, the ionization of water produces a huge amount of free radicals within milliseconds [6], which disrupt the structure and function of biomacromolecules, leading to immediate HSC apoptosis that peaks at hours post IR [4, 7]. Notably, these free radicals are also quenched within milliseconds [6], while the oxidative stress in HSCs is not relieved due to the deregulation of redox system [7]. Besides, metabolic regulation is a powerful intrinsic principle guiding HSC maintenance. Although homeostatic HSC metabolism is featured by glycolysis, after injury HSC activation usually accompanies extensive metabolic rewiring that is marked by augmented mitochondrial oxidative phosphorylation (OXPHOS), which fuels HSC proliferation but further poses oxidative and survival stresses to HSCs [8, 9]. As a result, HSCs surviving IR-induced immediate apoptosis still suffer from cell demise such as ferroptosis that peaks at and persists from 1 day post IR (dpi), which precludes their effective regeneration [5]. Hence, how HSCs deal with continuous cell demise represents a key node to understand the process of HSC regeneration and to search for effective therapeutic avenues against myelosuppression.

HSCs localize in a specialized microenvironment termed niche. The HSC niche consists of multiple cell types, including mesenchymal cells and HSC progenies such as megakaryocytes (MKs) and immune cells [2]. The niche plays essential roles in transmitting the signal of hematopoietic demand to HSCs and regulating HSC maintenance through intercellular signals in the form of cell-bound or secreted factors, or physical interaction [1, 2]. Unfortunately, myelosuppressive injury such as IR will profoundly disrupt the HSC niche, depleting and reorganizing the cellular niche of HSCs [10–12]. Of note, within the niche MKs are particularly resistant to myelosuppressive injury and remain functional in rodents for 7∼10 days after injury [13–15]. Meanwhile, MKs are recently demonstrated to have a very intimate interaction with HSCs, as they not only are the unique progeny known to be directly generated from HSCs [16], but also localize adjacent to HSCs and reciprocally regulate the maintenance of HSCs through paracrine signalings [17–22]. Logistically, it is reasonable to consider MKs as the most probable candidate for the source of regulatory signals in the HSC niche after myelosuppressive injury. Nevertheless, how MKs respond to myelosuppressive injury and their distinct roles in HSC regeneration remain largely undefined.

In this study, by using a mouse model of IR-induced myelosuppressive injury, we show that megakaryocytic IGF1 hypersecretion after myelosuppressive injury is a crucial niche signal for optimal HSC regeneration by promoting proliferation and restricting ferroptosis of HSCs. Meanwhile, punctual IGF1 administration represents an effective approach to promote HSC regeneration and recovery from myelosuppression. These findings not only identify megakaryocytic IGF1 hypersecretion as a protective mechanism evolved to safeguard HSC regeneration, but also offer a rational and safe medical countermeasure for myelosuppression.

## Methods

### Animals

C57BL/6 mice were purchased from Beijing HFK Bioscience Co., Ltd (Beijing, China). C57BL/6-Tg(Pf4-icre)Q3Rsko/J (*Pf4-Cre*) mice, B6.129(FVB)-*Igf1*^*tm1Dlr*^/J mice (*Igf1*^*f/f*^) and C57BL/6J-*Mpl*^*hlb219*^/J (*Mpl*^*hlb219*^) mice were purchased from the Jackson Laboratory. B6/JGpt-*Ptprc*^em1Cin(p.K302E)^/Gpt (CD45.1) mice were purchased from GemPharmatech (Nanjing, China). EGFP Reporter (GFP) mice were purchased from Cyagen Biosciences (Santa Clara, CA, USA). GFP-LC3 transgenic mice were kindly provided by professor Dengqun Liu (University of Electronic Science and Technology of China, Sichuan, China) [23]. Male transgenic mice and littermate controls were used in the experiments. All mice used were randomized, background-matched, and age-matched. All mice used were housed in specific pathogen-free conditions and fed with autoclaved water and food. Animal experiments were approved by the Animal Care Committee of the Army Medical University (NO. AMUWEC2019092) and conducted according to the institutional guidelines.

### Irradiation

To induce myelosuppressive injury, mice were exposed to a single dose of 5 Gy total body irradiation (TBI) with a ^60^Co γ-ray source in the Irradiation Center (Army Medical University, Chongqing, China). The dose rate of IR was 92.8 to 95.5 cGy/min.

### Pharmacological treatment

For IGF1 administration, mice were subcutaneously treated with a dose of 200 µg/kg recombinant mouse IGF1 (R&D Systems, Minneapolis, MN, USA). For rapamycin (RAPA) and imidazole ketone erastin (IKE) administration, mice were intraperitoneally treated with a dose of 4 mg/kg RAPA (MedChem Express, Monmouth Junction, NJ, USA) or 40 mg/kg IKE (MedChem Express).

### Hematological parameter test

Hematological parameters of mice were determined as we previously reported [5]. In brief, 20 µL PB were collected from the tail veins of mice and diluted in 1% EDTA solution, followed by being automatically counted by a Sysmex XT-2000i hematology analyzer (Sysmex Corporation, Kobe, Japan).

### Transplantation studies

For competitive repopulation assays, 5 × 10^5^ BMCs (CD45.2) from control or IGF1-treated mice, together with 5 × 10^5^ competitor BMCs (CD45.1), were transplanted into lethally irradiated (10 Gy) recipient mice (CD45.1). Before transplantation, debris and dead cells were excluded by FSC-SSC and DAPI staining. PB was collected from the tail veins of mice and PB reconstitution was analyzed using flow cytometry 16 weeks after transplantation, when the vast majority of leukocytes are reconstituted by HSCs [24].

### Flow cytometry and cell sorting

For HSC (Lineage^**–**^Sca-1^+^c-Kit^+^Flt3^–^CD34^–^) phenotypic analysis, BMCs were stained with Sca-1 (D7), c-Kit (2B8), CD34(RAM34), Flt3 (A2F10), and mature lineage marker mix [CD3e (145-2C11), B220 (RA3-6B2), Gr-1 (RB6.8C5), Mac-1 (M1/70), and Ter-119 (Ter119)] (all eBioscience, San Diego, CA, USA). For MK (CD41^+^CD42d^+^) phenotypic analysis, cells were stained with CD41 (MWReg30) and CD42d (1C2) antibody (all Biolegend, San Diego, CA, USA). Platelets were selected based on forward scatter (FSC) and side scatter (SSC) characteristic and CD41^+^ platelets were selected for analysis.

For MK subpopulations analysis, BMCs were stained with CD41 (MWReg30) antibody (Biolegend) and carefully washed. Cells were firstly fixed with IC Fixation buffer (eBioscience) at room temperature for 20 min and then permeabilized with Permeabilization buffer (eBioscience) in the presence of anti-MYLK4, anti-LSP1 and anti-ARNTL antibodies (all Abmart, Shanghai, China) at room temperature for another 30 min. Cells were then stained with fluorescent dye conjugated secondary antibodies (Thermo Fisher Scientific, Carlsbad, CA, USA) and finally analyzed by flow cytometry. MKs were identified as nMK (CD41^+^MYLK4^+^), iMK (CD41^+^LSP1^+^) and pMK (CD41^+^ARNTL^+^). MkPs was then calculated by stacking percentage after excluding the before-mentioned components.

For phenotypic analysis of BM cells, cells were stained with a combination of fluorochrome-conjugated antibodies, including CD45 (30-F11), CD3e (145-2C11), B220 (RA3-6B2), CD80 (B7-1), Ter-119 (Ter119), CD11b (M1/70), CD11c (N418), CD41a (eBioMWReg30), CD31 (390), and CD51 (RMV-7) (all eBioscience) antibodies. BMCs were defined as follows: B cell (CD45^+^B220^+^), T cell (CD45^+^CD3e^+^), mono (CD45^+^CD80^**–**^CD11b^+^), Dc (CD45^+^CD11c^+^), endo (CD45^–^Ter119^–^CD31^+^), MSC (CD45^–^Ter119^–^CD31^–^CD51^+^), and other hematopoietic cells described as above.

Intracellular protein expression detection was performed as we previously reported [25]. Briefly, BMCs were stained with HSC markers and carefully washed. Cells were firstly fixed with IC Fixation buffer (eBioscience) at room temperature for 20 min and then permeabilized with Permeabilization buffer (eBioscience) in the presence of anti-phospho-AKT^Ser473^ (eBioscience), anti-phospho-mTOR^Ser2448^ (eBioscience), anti-IGF1 (Thermo Fisher Scientific), and anti-phospho-IGF1R^Tyr1131^ (BD Phosflow, San Jose, CA, USA), at room temperature for another 30 min. If necessary, cells were stained with fluorescent dye conjugated secondary antibodies (Thermo Fisher Scientific) and finally analyzed by flow cytometry.

For isolation of MKs and HSCs, BMCs were incubated in PBS + 2% FCS for 20 min with a Direct Lineage Cell Depletion Cocktail (Miltenyi Biotec, Bergisch Gladbach, Germany) at 4 °C. Lineage^**–**^ cells were enriched by subsequent magnetic cell separation. Cells were sorted using a FACSAriaII or analyzed using a LSRFortessa (all BD Biosciences, San Jose, CA, USA) flow cytometer. The antibodies used are listed in Supplementary Table 1. All flow cytometry data was analyzed using FlowJo V10 software (Treestar Inc., San Carlos, CA, USA).

### Cell cycle analysis

BMCs were stained with indicated surface markers and carefully washed. 1 mL of Foxp3 Fixation/Permeabilization working solution was added to the resuspended cells and then incubated at room temperature for 30 min. Then, the cells were permeabilized with Permeabilization working solution in the presence of an anti-Ki-67 (eBioscience) antibody at room temperature for 30 min. After careful wash, cells were incubated with 0.1 mg/mL DAPI (Biolegend) for another 15 min at room temperature, followed by analysis with a flow cytometer.

### Mitochondrial assay

For detecting mitochondrial function, pre-stained BMCs were suspended in 1 mL prewarmed (37 °C) Flow Cytometry Staining Buffer with 30 nM Mito-Tracker Green (MTG) or 100 nM tetramethylrhodamine ethyl ester (TMRE), (all Thermo Fisher Scientific), together with 50 µM verapamil [26] (Sigma-Aldrich, St. Louis, MO, USA). After been incubated at 37 °C for 30 min, cells were washed twice and immediately analyzed with a flow cytometer.

### Adoptive MK transfer

For adoptive transfer of BM MKs, GFP mice (donor) and *Mpl*^*hlb219*^ mice (recipient) were simultaneously exposed to a single dose of 5 Gy TBI. Then, 3** × **10^5^ BM MKs from GFP mice, *Igf1*^*f/f*^ or *Igf1*^*ΔMK*^ mice at 2 dpi were obtained by continuously enriching CD41^+^ cells and CD42d^+^ cells using an EasySep™ Release Mouse Biotin Positive Selection Kit and an EasySep™ Release Mouse PE Positive Selection Kit (all StemCell Technologies, Vancouver, BC, Canada) with biotin-labeled anti-CD41 and PE-labeled anti-CD42d antibodies according to the manufacturer’s instructions. Subsequently, the freshly-isolated MKs were intravenously transfused into *Mpl*^*hlb219*^ mice.

### Immunofluorescence

Femurs and tibias from indicated mice were isolated and fixed for 24 h at room temperature with 4% paraformaldehyde (PFA; Affymetrix, Cleveland, OH, USA) and subsequently bisected along the long axis to expose the BM cavity. Bones were post-fixed for 30 min with the same fixation solution stated above and blocked with 10% goat serum (Thermo Fisher Scientific) for 30 min. For analysis of anatomical distances of MK**s** to HSCs, the slides were successively stained with anti-mouse CD150 (TC15-12F12.2), biotin-conjugated anti-mouse CD48 (HM48-1), anti-IGF1 (2872R), and biotin-conjugated anti-mouse Lineage cocktail (TER-119, RB6-8C5, RA3-6B2, M1/70, 145-2C11) antibodies as indicated at 4℃ overnight. Subsequently, the slides were incubated with Pacific Blue Conjugate Streptavidin **(**Thermo Fisher Scientific**)** and corresponding fluorescent secondary antibody for 1 h at room temperature. Imaging was performed using a Zeiss LSM800 NLO confocal microscope (Carl Zeiss, Jena, Germany). ZEN software (Carl Zeiss) was used to identify CD150^+^CD48^–^Lineage^**–**^ HSCs and MKs identified by morphology, and the distance between cell centers was calculated. Data were collected in an automated blinded fashion. To detect HSC mitochondria, freshly FACS-sorted HSCs were stained with TOMM20 (Santa Cruz Biotechnology) antibody (37 °C, 30 min) and placed onto poly-L-lysine coating slides with 10 µL HBSS (Gibco, Grand Island, NY, USA). Then, the sections were stained with appropriate fluorescent dye conjugated secondary antibodies (Thermo Fisher Scientific). Finally, the sections were counterstained with DAPI and photographed as soon as possible.

### RNA-seq

HSCs were sorted from mouse BM, and RNA was extracted using a RNeasy Micro Kit **(**QIAGEN, Hilden, Germany**)** following the manufacturer’s instructions. The amount and purity of RNA were quantified using NanoDrop ND-1000 (Thermo Fisher Scientific). Library construction and RNA-seq were performed on illumina Novaseq™ 6000. Fastp software (https://github.com/OpenGene/fastp) was used to remove the adaptor contamination, undetermined bases, and low-quality bases. After sequence alignment, gene expression was quantified by RSEM (version: v1.2.12, http://deweylab.biostat.wisc.edu/ RSEM) and differential gene expression (DEG) evaluation was analyzed by DEseq2 using a fold change > 2.00 and adjusted *P* value < 0.05. Gene set enrichment analysis (GSEA, Broad Institute) was performed based on Molecular Signatures Database v6.0 (http://software.broadinstitute.org/gsea/msigdb). Ingenuity Pathway Analysis (QIAGEN) was used to analyze signaling pathways. Enrichment of pathway specific gene sets was performed using PANTHER using Reactome version 86.

### Transcriptomic analysis of BMCs and MKs

The *Igf1* expression was reanalyzed using published transcriptomic datasets [27, 28] with authors’ permissions. The transcriptomic signature of MK subpopulation was established using scRNA-seq datasets [29] with authors’ permissions.

### MK heterogeneity analysis

MK heterogeneity analysis was processed based on the published scRNA-seq datasets of MK. Briefly, differential expression genes (DEGs) of four MK subpopulations were filtered with power > 0.4 and “avg _diff” >0.7. Subsequently, signatures of MK subpopulation genes were identified with myAUC > 0.8. (power: the predictive power; ave_diff: the Log fold change of average expression in the current cluster against the rest of clusters; myAUC: the area under the ROC curve.) RNA-seq data of MKs post IR were reanalyzed with signatures of MK subpopulations for mapping the functional alterations. After standardizing the data, up-regulated and down-regulated genes were displayed by heatmap. In order to quantitatively evaluate MK subset alterations, top five signatures of MK subpopulation genes were filtered. After Z-Score normalization and combination, signatures of MK subpopulation were presented in the radar plot.

### MK ploidy analysis

MK ploidy analysis was performed as we previously reported [30]. Cells were fixed in 75% ethanol for 24 h and then cells were labeled with anti-CD41 for 30 min at 4 ℃. Finally, cells were treated with RNase (Sigma-Aldrich) followed by 20 µg/ml prodium iodide (PI, Sigma-Aldrich) to stain DNA for 30 min in the dark and then analyzed using Accuri C6 (BD Biosciences). Ploidy was measured in the CD41^+^ cell population.

### HSC culture

HSC culture was performed according to the method as previously reported, which efficiently maintains functional HSCs ex vivo [31]. Briefly, sorted HSCs were plated in 24-well fibronectin-coated plate and cultured in medium composed of F12 medium, 1% transferrin, 1% penicillin/streptomycin/glutamine, 10 mM HEPES (all Gibco), IGF1 (10 ng/mL), SCF (10 ng/mL), TPO (100 ng/mL; all PeproTech, Rocky Hill, NJ, USA), and polyvinyl alcohol (PVA; 1 mg/mL, Sigma-Aldrich) at 37℃ and 5% CO_2_. Cell numbers per well were counted every other day using a hematocytometer (Countess™ II FL; Thermo Fisher Scientific) and dead cells were excluded by trypan blue. For ferroptosis resistance assay, HSCs were cultured in in the presence of 5 µM erastin (MedChem Express) for 48 h.

### HSC co-culture

HSCs and MKs were FACS-sorted from mice at 3 dpi. 1 × 10^3^ HSCs were cocultured with 1 × 10^4^ BM MKs (CD41^+^CD42d^+^) or 1 × 10^4^ other niche cells (CD41^−^CD42d^−^) in the culture medium described above. 10 µM AG1024 (MedChem Express) was added in the culture in the indicated group. Supernatant of the culture was collected for IGF1 measurement at day 3, while HSC phenotype and colony assay was processed at day10 post coculture.

### Colony assay

Colony assay was performed as we previously reported [10]. 1000 sorted Lineage^**–**^ cells from HSC cultures were plated into methylcellulose medium (STEMCELL Technologies) according to the manufacturer’s protocols. Colonies were assessed after 12 days using an inverted light microscopy (Thermo Fisher Scientific).

### Intracellular total iron and ATP measurement

Freshly FACS-sorted HSCs were rinsed with PBS and carefully collected with centrifugation. Intracellular total iron and ATP contents in HSCs were respectively measured by an Iron Assay Kit (Abcam) and a Luminescent ATP Detection Assay Kit (Abcam) according to the manufacturer’s instructions. Data were normalized to the total protein level in each sample.

### Ferroptosis assay

For ferroptosis analysis, BMCs were firstly stained with HSC markers and carefully washed. Cell death analysis was performed by suspending cells in a 7-amino-actinomycin D (7-AAD) staining solution (eBioscience) and incubating for 15 min at room temperature. Lipid peroxidation was measured by suspending cells in prewarmed (37 °C) PBS with 10 µM Liperfluo (Dojindo Molecular Technologies, Kumamoto, Japan) and incubating for 30 min at 37 °C. Ferrous ion deposition was measured by suspending cells in prewarmed (37 °C) PBS with 2 µM FerroOrange (Dojindo Molecular Technologies) and incubating for 30 min at 37 °C.

### Ferritinophagy assay

For ferritinophagy analysis, BMCs of GFP-LC3 mice were stained with HSC markers and followed by flow cytometric detection of GFP-LC3. For ferritin detection, cells were permeabilized with Permeabilization buffer (eBioscience) in the presence of anti-Ferritin (Abcam) antibody at room temperature for 45 min and stained with fluorescent dye conjugated secondary antibodies (Thermo Fisher Scientific) at room temperature for another 30 min and finally analyzed by flow cytometry.

### Quantitative RT-PCR (qRT-PCR)

RNA was extracted from FASC-sorted MKs using a RNeasy® Micro Kit (QIAGEN, Hilden, Germany) and was reverse-transcribed into cDNA using a PrimeScript™ RT reagent Kit (TaKaRa, Shiga, Japan) according to the manufacturer’s instructions. The mRNA expression levels of indicated genes were measured by a CFX96™ Real-Time system (BioRad, Hercules, CA, USA) with a GoTaq® qPCR Master Mix (Promega, Madison, WI, USA). The primers were listed in Supplementary Table 2.

### IGF1 measurement

IGF1 level in the plasma and BM fluid was measured by Mouse IGF1 ELISA Kit (R&D systems) according to the manufacturer’s instructions.

### Statistical analysis

Statistical analysis was performed using Prism 9.0 (GraphPad Software, USA). All results are expressed as mean ± standard deviation (SD). *n* represents the number of independent experiments, as detailed in figure legends. Comparisons between two groups were determined by paired or unpaired two-tailed Student’s *t*-test. Three or more groups were compared by one-way analysis of variance (ANOVA) followed by Tukey-Kramer post hoc analysis. *P* < 0.05 was considered statistically significant. The vast majority of ex vivo experiments have been performed multiple times. Most in vivo experiments have been performed at least twice.

## Results

### MKs become a predominant component and IGF1 source of HSC niche after radiation injury

**Fig. 1:**
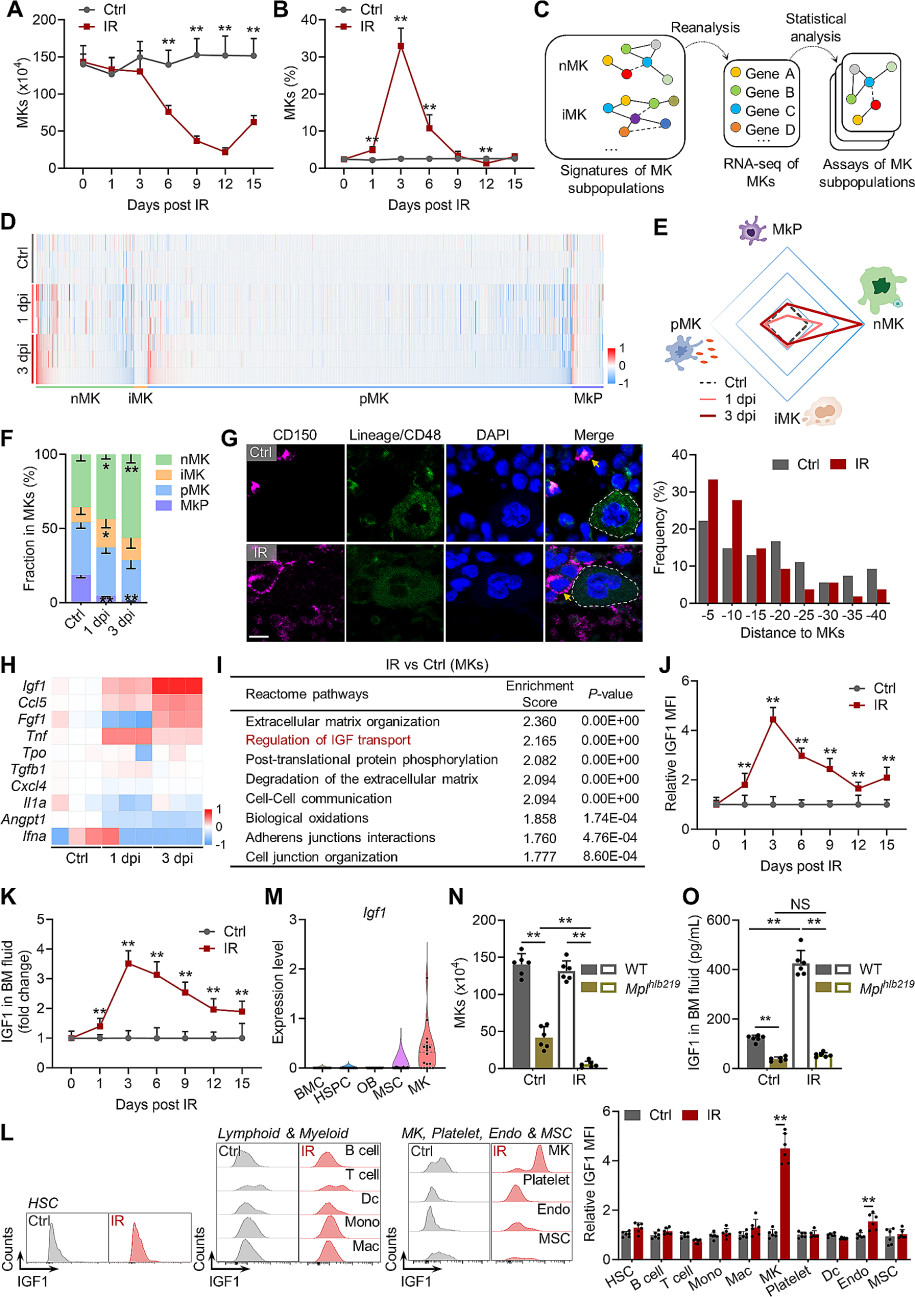
MKs become a predominant component and IGF1 source of HSC niche after radiation injury. (**A** and **B**) The number and frequency of MKs in the BM of mice at indicated time post IR (*n* = 6). (**C**) Schematic illustration of MK subpopulation assay. (**D**) Heatmap showing the expression of genes related to each MK subpopulation at indicated time post IR. (**E**) Radar plot showing functional shift of MKs at indicated time post IR. (**F**) Flow cytometric analysis of the fraction of MK subpopulations in the BM of mice at indicated time post IR (*n* = 6). (**G**) Immunostaining analysis of positional relationship between HSCs and MKs in the BM of mice at 3 dpi. The yellow arrow indicates HSC. The dashed line outlines MK. Scale bar, 10 μm (*n* = 60). (**H**) Heatmap showing the expression of megakaryocytic secretory factors at indicated time post IR. (**I**) Reactome pathway enrichment analysis of BM MKs at 3 dpi. (**J**) Flow cytometric analysis of IGF1 expression in BM MKs of mice at indicated time post IR (*n* = 6). (**K**) Relative IGF1 levels in the BM fluid of mice at indicated time post IR (*n* = 6). (**L**) Flow cytometric analysis of IGF1 expression in BMC and platelets of mice at 3 dpi. Mono, monocyte; Mac, macrophage; Dc, dendritic cell; Endo, endothelium; MSC, mesenchymal stromal cell. (*n* = 6). (**M**) Violin plots showing *Igf1* expression in different cell clusters at homeostasis. Hematopoietic stem and progenitor cell, HSPC; osteoblast, OB. (**N**) MK numbers in the BM of WT and *Mpl*^*hlb219*^ mice at 3 dpi (*n* = 6). (**O**) Relative IGF1 levels in the BM fluid of WT and *Mpl*^*hlb219*^ mice at 3 dpi (*n* = 6). Data represent mean ± SD. *P < 0.05, **P < 0.01, NS: no significance. Two-tailed unpaired student’s *t*-test unless stated otherwise. One-way ANOVA was used for calculating *P* values in (**F**), (**N**) and (**O**)

Initially, we created a mouse model of myelosuppressive injury by exposing mice to a sublethal dose of IR. The dynamic changes in the number, distribution and functionality of BM MKs were monitored during the early post-IR interval. Consistently, unlike the vast majority of BMCs (Supplementary Fig. 1A), MKs were resistant to IR as they were scarcely eliminated by 3 dpi (Fig. 1A). Consequently, the frequency of MKs dramatically increased post IR, reaching approximately 30% at 3 dpi (Fig. 1B). Recent studies unveiled that the MK population can be classified into MK progenitors (MkPs) as well as subpopulations that exert functions of niche support (nMK), immunity (iMK), and platelet production (pMK), which are purported to be distinguished by the expression of MYLK4, LSP1 and ARNTL, respectively [29, 32, 33]. By comparing the expression of the transcriptional signature of each MK subpopulation in IR versus control mice (Fig. 1C and D), we observed an outstanding functional shift towards nMKs post IR (Fig. 1E), which was further validated by flow cytometry (Fig. 1F, Supplementary Fig. 1B). Meanwhile, the proportion of high-ploidy (8–32 N) MKs, which are reported to enrich for nMKs [21, 29], was remarkably increased after IR (Supplementary Fig. 1C). In support of the overrepresentation of nMKs, histological analysis showed that MKs got much closer to HSCs in the BM of IR versus control mice (Fig. 1G).

Niche MKs principally regulate HSC homeostasis through paracrine of cytokines [34]. When analyzing the secretion profile of MKs, we detected that the upregulation of IGF1 was most prominent post IR (Fig. 1H). Pathway enrichment analysis confirmed that IGF1 secretion was predominantly activated in MKs post IR (Fig. 1I). Flow cytometry also detected dramatically increased IGF1 secretion in MKs after IR (Fig. 1J), along with parallelly increased IGF1 levels in the BM rather than in the plasma, which peaked at 3 dpi (Fig. 1K, Supplementary Fig. 1D). Intriguingly, among the tissues we noticed that IGF1 expression was exclusively upregulated in the BM (Supplementary Fig. 1E). Among BMCs including hematopoietic, immune and mesenchymal cells, IGF1 secretion was almost exclusively augmented in MKs, particularly in the high-ploidy nMKs, while that was unchanged in platelets at 3 dpi (Fig. 1L, Supplementary Fig. 1F and G). Moreover, basal IGF1 secretion seemed to be strongest in MKs within the BM based on the published RNA-seq datasets [27, 28] (Fig. 1M, Supplementary Fig. 1H). Using a mouse model of defective megakaryopoiesis which harbors a homozygous mutation of the TPO receptor (*Mpl*^*hlb219*^), we found that MKs were nearly absent and the BM IGF1 levels were only marginally elevated in *Mpl*^*hlb219*^ mice post IR (Fig. 1N and O). These data indicate that MKs serve as a predominant source of IGF1 in the BM after radiation injury. Given that IGF1 has been identified as a marker gene of nMKs [29, 35], the overrepresentation of nMKs and the predominant IGF1 upregulation in nMKs may collectively lead to the preferential IGF1 hypersecretion by surviving MKs after radiation injury.

### Megakaryocytic IGF1 favors HSC maintenance after radiation injury

**Fig. 2:**
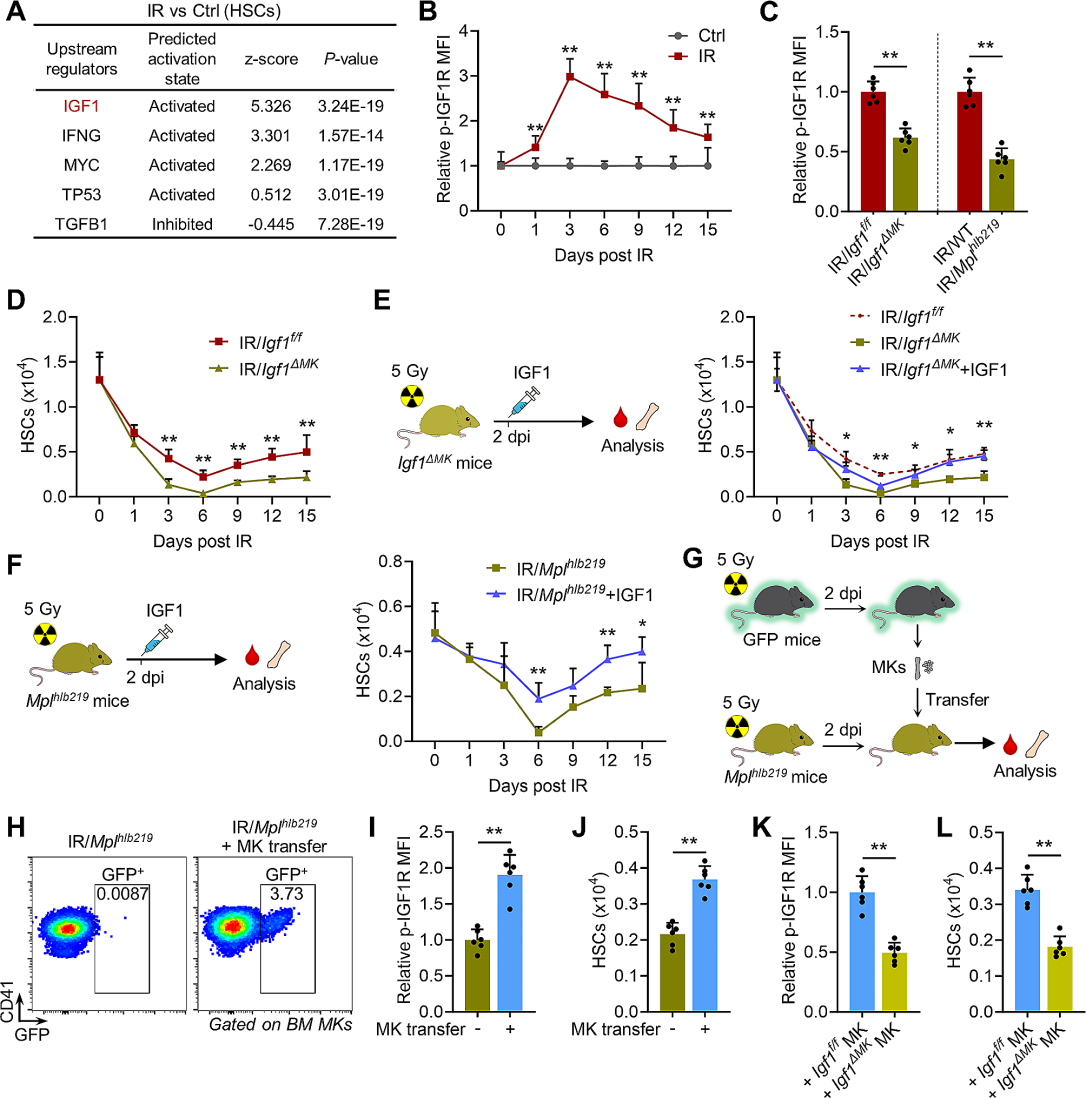
Megakaryocytic IGF1 favors HSC maintenance after radiation injury. (**A**) IPA Upstream regulators prediction analysis of BM HSCs from mice at 3 dpi (IR vs. control). (**B**) Flow cytometric analysis of p-IGF1R expression in HSCs in the BM of mice at indicated time post IR (*n* = 6). (**C**) Flow cytometric analysis of p-IGF1R expression in HSCs in the BM of *Igf1*^*ΔMK*^ and *Mpl*^*hlb219*^ mice at 3 dpi (*n* = 6). (**D**) The number of HSCs in the BM of *Igf1*^*f/f*^ and *Igf1*^*ΔMK*^ mice at indicated time post IR (*n* = 6). (**E**) Experimental design and the number of HSCs in the BM of *Igf1*^*f/f*^ and *Igf1*^*ΔMK*^ mice with or without IGF1 supplementation at indicated time post IR (*n* = 6). (**F**) Experimental design and the number of HSCs in the BM of *Mpl*^*hlb219*^ mice with or without IGF1 supplementation at indicated time post IR (*n* = 6). (**G**) Experimental design of adoptive MK transfer. (**H**) Flow cytometric analysis of frequency of donor-derived GFP^+^ MKs in the BM of *Mpl*^*hlb21****9***^ mice with or without adoptive MK transfer at 3 dpi (*n* = 6). (**I** and **J**) Flow cytometric analysis of p-IGF1R expression in HSCs and the number of HSCs in the BM of *Mpl*^*hlb219*^ mice with or without MK transfer at 3 dpi (*n* = 6). (**K** and **L**) Flow cytometric analysis of p-IGF1R expression in HSCs and the number of HSCs in the BM of *Mpl*^*hlb219*^ mice with *Igf1*^*f/f*^ or *Igf1*^*ΔMK*^ mice-derived MK transfer at 3 dpi (*n* = 6). Data represent mean ± SD. *P < 0.05, **P < 0.01. Two-tailed unpaired student’s *t*-test. *Igf1*^*ΔMK*^ mice with or without IGF1 supplementation at indicated time post IR was used for calculating *P* values in (**E**)

The functional preference towards nMKs post IR inferred a close link to the modulation of HSC maintenance. In favor of this, prediction of upstream regulatory factors by Ingenuity Pathway Analysis of HSC transcriptomes revealed predominantly activated IGF1 signaling in HSCs of IR versus control mice at 3 dpi (Fig. 2A). Flow cytometry verified that activation of IGF1R on HSCs was initiated from 1 dpi and peaked at 3 dpi (Fig. 2B, Supplementary Fig. 2A), paralleling the dynamics of megakaryocytic IGF1 hypersecretion (Fig. 1J). To elucidate the underlying pathophysiological significance, we conditionally deleted *Igf1* from MKs by using *Pf4-Cre*/*Igf1*^*f/f*^ (*Igf1*^*ΔMK*^) mice (Supplementary Fig. 2B). Unlike *Mpl*^*hlb219*^ mice, in which BM HSC maintenance and hematopoiesis were impaired (Supplementary Fig. 2C), *Igf1*^*ΔMK*^ mice exhibited nearly unaffected BM HSC maintenance and hematopoiesis at steady state as compared to littermate controls (Supplementary Fig. 2D and E), consistent with previous studies [36, 37]. These data inform that megakaryocytic IGF1 is dispensable for homeostatic HSC maintenance. Correspondingly, after myelosuppressive injury IGF1 signaling activation in HSCs was significantly dampened in both *Igf1*^*ΔMK*^ mice and *Mpl*^*hlb219*^ mice at 3 dpi (Fig. 2C). Meanwhile, although megakaryocytic IGF1 deficiency had minimal impact on acute HSC ablation at 1 dpi, it further reduced the HSC pool size from 3 dpi (Fig. 2D), accompanied by remarkably exacerbated myelosuppression (Supplementary Fig. 2F). However, supplementation of a single dose of recombinant mouse IGF1 at 2 dpi significantly reverted these detrimental effects in *Igf1*^*ΔMK*^ mice (Fig. 2E, Supplementary Fig. 2G and H). With regard to the leukocyte compartment, IGF1 supplementation accelerated the recovery of both myeloid and lymphoid lineages in *Igf1*^*ΔMK*^ mice post IR (Supplementary Fig. 2I and J). Moreover, IGF1 supplementation also significantly augmented IGF1 signaling activation (Supplementary Fig. 2K), increased HSC pool size (Fig. 2F) and attenuated myelosuppression (Supplementary Fig. 2L) in *Mpl*^*hlb219*^ mice post IR. Furthermore, IR-experienced MKs were adoptively transferred into *Mpl*^*hlb219*^ mice post IR and they successfully lodged to the BM and localized adjacent to HSCs (Fig. 2G and H, Supplementary Fig. 2M). Similarly, the IGF1 signaling activation and maintenance of HSCs as well as hematopoietic recovery in *Mpl*^*hlb219*^ mice post IR were significantly enhanced by MK adoptive transfer (Fig. 2I and J, Supplementary Fig. 2N), while these effects were nearly vanished by adoptive transfer of MKs from *Igf1*^*ΔMK*^ mice (Fig. 2K and L, Supplementary Fig. 2O). These data indicate that megakaryocytic IGF1 hypersecretion favors HSC maintenance after radiation injury.

### Megakaryocytic IGF1 promotes functional expansion of HSCs after radiation injury

**Fig. 3:**
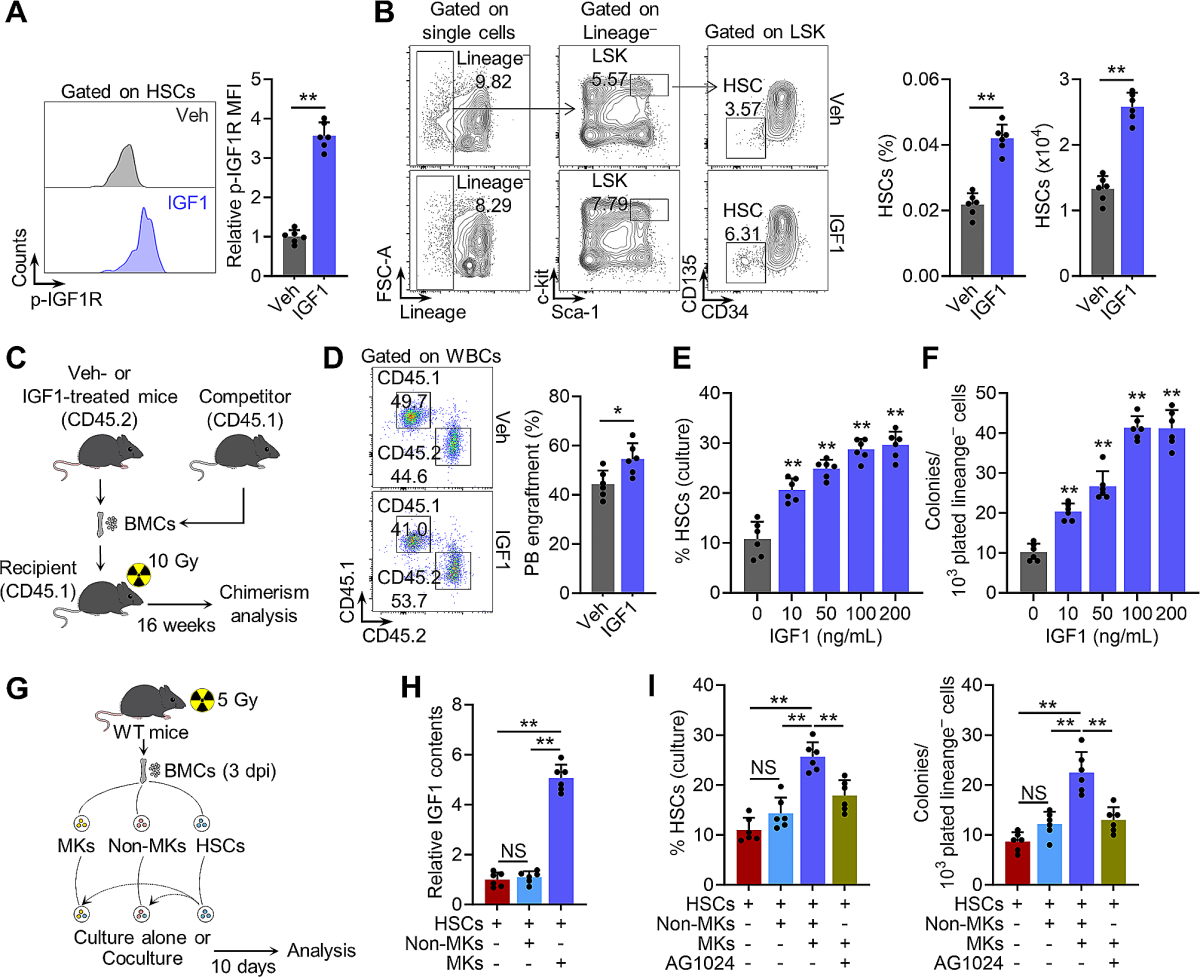
Megakaryocytic IGF1 promotes functional expansion of HSCs after radiation injury. (**A**) Flow cytometric analysis of p-IGF1R expression in HSCs of mice at day 1 post IGF1 administration (*n* = 6). (**B**) Flow cytometric gating strategy, frequency and the number of HSCs in the BM of mice at day 1 post IGF1 administration (*n* = 6). (**C** and **D**) Experimental design and PB chimerism at 16 weeks post-competitive transplantation of HSCs from mice at day 1 post IGF1 administration (*n* = 6). (**E**) Frequencies of HSCs in the ex vivo culture system with different concentrations of IGF1 at day 10 (*n* = 6). (**F**) Colony numbers of per 10^3^ lineage^**–**^ cells sorted from HSC cultures as indicated (*n* = 6). (**G**) Experimental design of an ex vivo co-culture experiment. (**H**) Relative IGF1 contents in the supernatant of the indicated culture (*n* = 6). (**I**) Frequencies of HSCs and colony numbers of per 10^3^ lineage^**–**^ cells sorted from various HSC cultures. 10 µM AG1024 was added in the culture in the indicated group (*n* = 6). Data represent mean ± SD. *P < 0.05, **P < 0.01, NS: no significance. One-way ANOVA unless stated otherwise. Two-tailed unpaired student’s *t*-test was used for calculating *P* values in (**A**), (**B**) and (**D**)

Chronic activation of IGF1 signaling is well known to negatively regulate HSC maintenance [27, 38], while the effect of transient IGF1 stimulation such as that observed post IR on HSC maintenance is undefined. To this end, mice were administered with a single dose of IGF1 to mimic transient IGF1 hypersecretion in vivo. One day after IGF1 administration, we detected remarkable activation of IGF1 signaling and expansion of HSCs (Fig. 3A and B). Competitive transplantation verified that the donor chimerism of BMCs from IGF1-treated mice was significantly higher than that from control mice 16 weeks after transplantation (Fig. 3C and D), reflecting enhanced self-renewal capacity and functional expansion of HSCs in response to transient IGF1 secretion. Three days after IGF1 administration, the counts of WBCs, RBCs and platelets were all significantly increased in PB (Supplementary Fig. 3A), reinforcing that IGF1 acted at the level of multipotential HSCs. To further test the direct effect of IGF1 on HSCs, we exploited an ex vivo culture system that sustains HSC self-renewal and expands functional HSCs [31], and modified it by replacing insulin with IGF1. It was found that IGF1 dose-dependently promoted self-renewal and functional expansion of HSCs in vitro (Fig. 3E and F). Analogously, by using an ex vivo co-culture system (Fig. 3G), we observed that MKs from IR mice not only contributed to IGF1 hypersecretion (Fig. 3H) but also significantly promoted self-renewal and functional expansion of HSCs (Fig. 3I), while other niche cells (non-MKs) from IR mice failed to have such effects (Fig. 3I). Inversely, blockade of IGF1 signaling by a selective IGF1R inhibitor AG1024 nearly abolished the pro-expansive action of MKs (Fig. 3I). These lines of evidence point to that HSCs are particularly susceptible to transient IGF1 hypersecretion, and that megakaryocytic IGF1 promotes self-renewal and functional expansion of HSCs after radiation injury.

### mTOR orchestrates HSC activation in response to megakaryocytic IGF1 after radiation injury

**Fig. 4:**
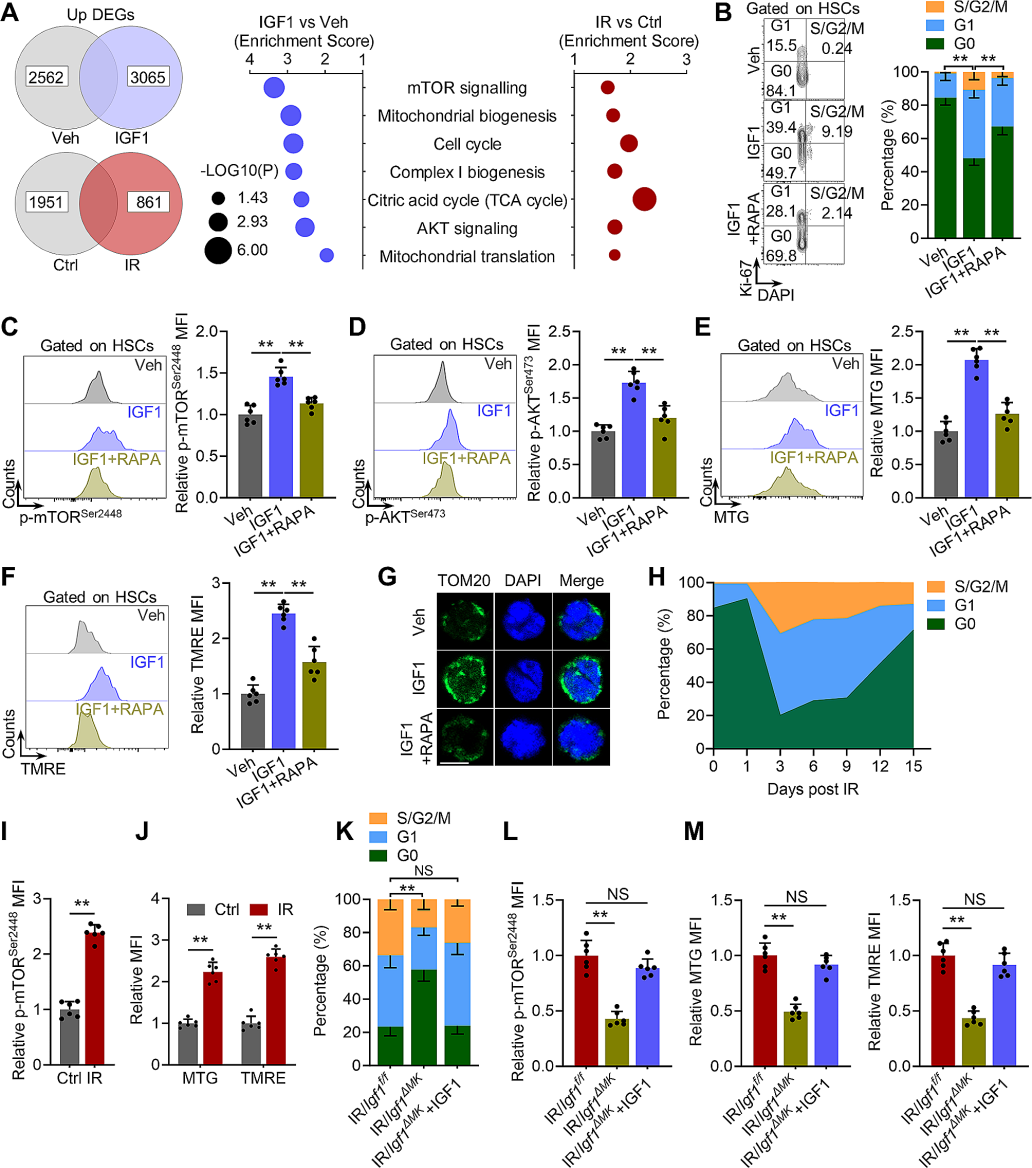
mTOR orchestrates HSC activation in response to megakaryocytic IGF1 after radiation injury. (**A**) Reactome pathways enrichment analysis of commonly upregulated signaling pathways in BM HSCs of mice at day 1 post IGF1 administration (IGF1 vs. vehicle) and at 3 dpi (IR vs. control). (**B**) Flow cytometric analysis of cell cycle of HSCs in the BM of mice at 1 day post IGF1/RAPA administration (*n* = 6). (**C** and **D**) Flow cytometric analysis of expression of p-mTOR and p-AKT in HSCs in BM of mice at 1 day post IGF1/RAPA administration (*n* = 6). (**E** and **F**) Flow cytometric analysis of MTG and TMRE in HSCs in BM of mice at 1 day post IGF1/RAPA administration (*n* = 6). (**G**) Immunostaining analysis of HSC mitochondria of mice at 1 day post IGF1/RAPA administration. Scale bar, 5 μm. (**H**) Flow cytometric analysis of cell cycle of HSCs in the BM of mice at indicated time post IR (*n* = 5). (**I**) Flow cytometric analysis of p-mTOR expression in HSCs in the BM of mice at 3 dpi (*n* = 6). (**J**) Flow cytometric analysis of MTG and TMRE in HSCs in the BM of mice at 3 dpi (*n* = 6). (**K-M**) Flow cytometric analysis of cell cycle, p-mTOR expression, MTG and TMRE in HSCs in the BM of *Igf1*^*f/f*^ and *Igf1*^*ΔMK*^ mice with or without IGF1 supplementation at 3 dpi (*n* = 6). Data represent mean ± SD. **P < 0.01, NS: no significance. One-way ANOVA unless stated otherwise. Two-tailed unpaired student’s *t*-test was used for calculating *P* values in (**I**) and (**J**)

To understand the cellular and molecular basis for functional HSC expansion induced by transient IGF1 hypersecretion, we firstly enriched the top altered signaling pathways based on comparative transcriptomic analysis of HSCs with or without transient IGF1 stimulation. In accordance with the effects of chronic IGF1 signaling activation [27, 38], HSCs with transient IGF1 stimulation exhibited a transcriptomic signature of HSC activation including mTOR signaling activation, proliferation, and augmented mitochondrial biogenesis and oxidative metabolism (Fig. 4A, Supplementary Fig. 4A), all of which were validated by flow cytometry (Fig. 4B-F), immunostaining (Fig. 4G) and intracellular ATP assay (Supplementary Fig. 4B). Inhibition of mTOR by rapamycin (RAPA) nearly abrogated these effects (Fig. 4B-G), confirming a central role of mTOR in transducing IGF1 signaling. Similarly, RNA-seq revealed that HSCs also exhibited an activation signature at 3 dpi (Fig. 4A, Supplementary Fig. 4A). Flow cytometric analysis confirmed the dramatically increased HSC proliferation started from 3 dpi (Fig. 4H), accompanied by remarkably increased mTOR activation (Fig. 4I) as well as augmented mitochondrial biogenesis and oxidative metabolism at 3 dpi (Fig. 4J, Supplementary Fig. 4C), paralleling the dynamics of megakaryocytic IGF1 hypersecretion. In *Igf1*^*ΔMK*^ and *Mpl*^*hlb219*^ mice, however, HSC activation was significantly blunted post IR (Fig. 4K-M, Supplementary Fig. 4D-H). In contrast, IGF1 supplementation nearly reverted these effects in *Igf1*^*ΔMK*^ and *Mpl*^*hlb219*^ mice (Fig. 4K-M, Supplementary Fig. 4D-H). Thus, megakaryocytic IGF1 triggers *bona fide* activation of IGF1 signaling that is orchestrated by mTOR in HSCs. As reported, IGF1 can promote self-renewal proliferation of a variety of stem cells [39–42], and HSCs also undergo self-renewal proliferation after myelosuppressive injury [2]. These lines of evidence suggest that megakaryocytic IGF1 may facilitate functional HSC expansion at least partially through promoting self-renewal proliferation of HSCs after radiation injury.

### Megakaryocytic IGF1 restricts HSC ferroptosis after radiation injury

**Fig. 5:**
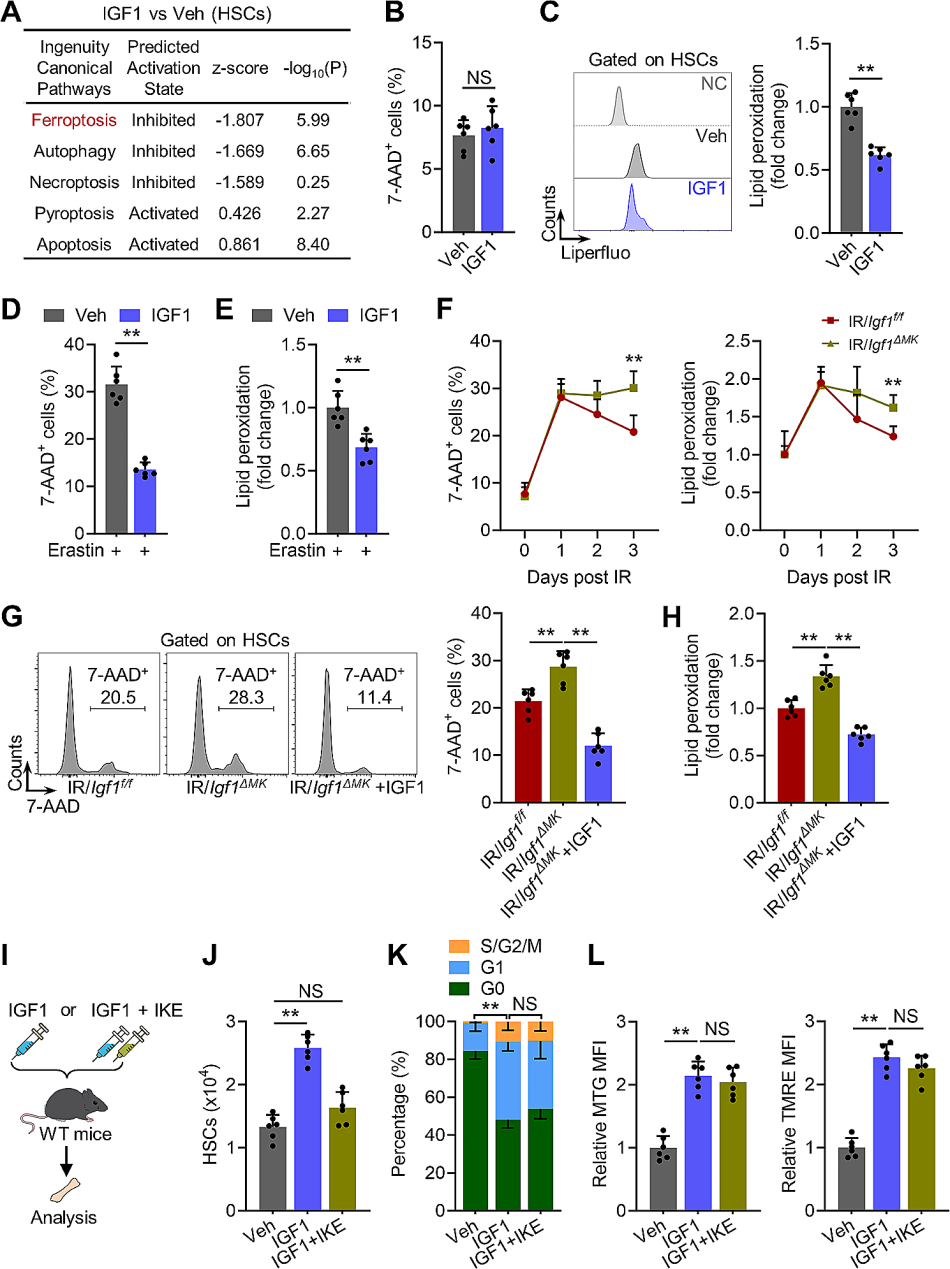
Megakaryocytic IGF1 restricts HSC ferroptosis after radiation injury. (**A**) IPA pathways enrichment analysis of BM HSCs of IGF1-treated mice (IGF1 vs. vehicle). (**B**) Frequency of cell death in HSCs in the BM of mice at 1 day post IGF1 administration (*n* = 6). (**C**) Flow cytometric analysis of lipid peroxidation of HSCs in BM of mice at 1 day post IGF1 administration (*n* = 6, NC: negative control). (**D** and **E**) Flow cytometric analysis of cell death and lipid peroxidation of BM HSCs from IGF1-treated mice in response to erastin (*n* = 6). (**F**) Flow cytometric analysis of cell death and lipid peroxidation of HSCs in the BM of *Igf1*^*f/f*^ and *Igf1*^*ΔMK*^ mice at indicated time post IR (*n* = 6). (**G** and **H**) Flow cytometric analysis of cell death and lipid peroxidation in HSCs in the BM of *Igf1*^*f/f*^ and *Igf1*^*ΔMK*^ mice with or without IGF1 supplementation at 3 dpi (*n* = 6). (**I**) Experimental design. (**J**) The number of HSCs in the BM of mice at 1 day post IGF1/IKE administration (*n* = 6). (**K** and **L**) Flow cytometric analysis of cell cycle, MTG and TMRE of HSCs in the BM of mice at 1 day post IGF1/IKE administration (*n* = 6). Data represent mean ± SD. **P < 0.01, NS: no significance. One-way ANOVA unless stated otherwise. Two-tailed unpaired student’s *t*-test was used for calculating *P* values in (**B-F**)

Notably, unlike other somatic stem cells, augmented proliferation and mitochondrial oxidative metabolism always culminates in HSC exhaustion due to HSC demise induced by replication stress and oxidative stress [8, 43], which can be illustrated by chronic IGF1 signaling activation [27, 38]. We further interrogated whether cell death and survival signaling pathways were reprogrammed in HSCs in response to transient IGF1 stimulation. Intriguingly, among the well-identified cell death signaling pathways, we noticed that ferroptosis signaling pathway was predominantly inhibited in HSCs of IGF1- versus vehicle-treated mice (Fig. 5A). In vivo, although HSC death in IGF1-treated mice was minimal and comparable with that in vehicle-treated mice at homeostasis (Fig. 5B), membrane lipid peroxidation, a well-recognized feature of ferroptosis, was significantly reduced in HSCs of IGF1-treated mice (Fig. 5C). Ex vivo, the freshly isolated HSCs from IGF1-treated mice were more resistant than those from vehicle-treated mice to ferroptosis induced by erastin, a widely-used ferroptosis inducer (Fig. 5D and E). IR is well demonstrated as an in vivo inducer of HSC ferroptosis, which peaked at 1 dpi and persisted for at least 3 days (Fig. 5F) [5]. Although HSC ferroptosis was indistinguishable in *Igf1*^*ΔMK*^ and *Mpl*^*hlb219*^ mice from that in their control counterparts at homeostasis and at 1 dpi, it was significantly exacerbated at 3 dpi (Fig. 5F, Supplementary Fig. 5A). Notably, the aggravated HSC ferroptosis in *Igf1*^*ΔMK*^ and *Mpl*^*hlb219*^ mice was significantly reverted by IGF1 supplementation (Fig. 5G and H, Supplementary Fig. 5B). Additionally, administration of an in vivo ferroptosis inducer IKE nearly abrogated HSC expansion without affecting HSC proliferation and mitochondrial oxidative metabolism after transient IGF1 stimulation (Fig. 5I-L, Supplementary Fig. 5C). These results demonstrate that megakaryocytic IGF1 restricts HSC ferroptosis after radiation injury, which may synergize with self-renewal proliferation to ensure functional HSC expansion.

### Megakaryocytic IGF1 inhibits ferritinophagy in HSCs via mTOR after radiation injury

**Fig. 6:**
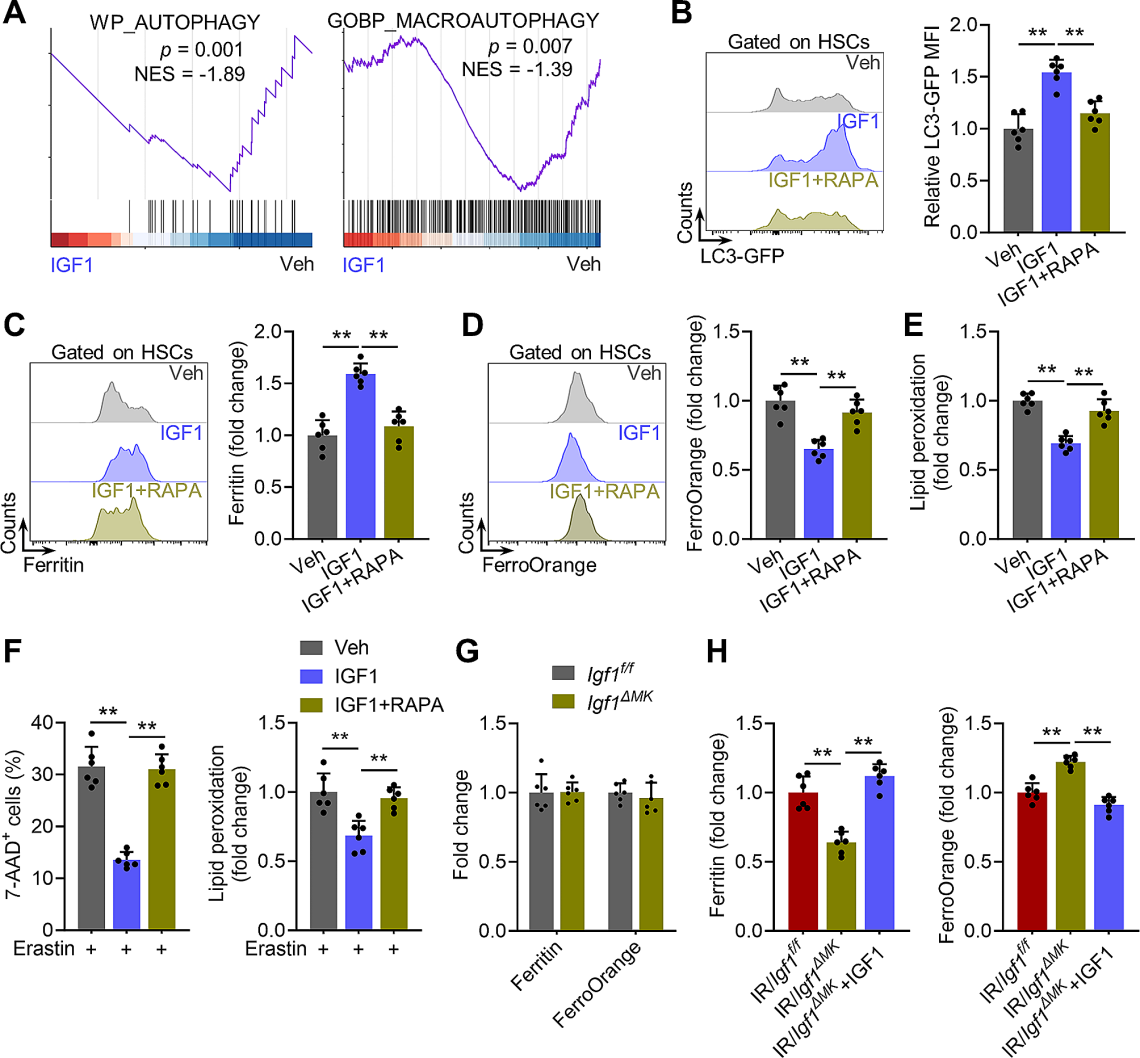
Megakaryocytic IGF1 inhibits ferritinophagy in HSCs via mTOR after radiation injury. (**A**) GSEA of the autophagy and macro-autophagy gene set in BM HSCs from mice at 1 day post IGF1 administration (IGF1 vs. vehicle). (**B**) Flow cytometric analysis of GFP-LC3 expression in HSCs in the BM of GFP-LC3 mice at 1 day post IGF1/RAPA administration (*n* = 6). (**C-E**) Flow cytometric analysis of Ferritin, FerroOrange and lipid peroxidation in HSCs in the BM of mice at 1 day post IGF1/RAPA administration (*n* = 6). (**F**) Flow cytometric analysis of cell death and lipid peroxidation in HSCs from IGF1/RAPA-treated mice in response to erastin (*n* = 6). (**G**) Flow cytometric analysis of Ferritin and FerroOrange in HSCs in the BM of *Igf1*^*f/f*^ and *Igf1*^*ΔMK*^ mice (*n* = 6). (**H**) Flow cytometric analysis of Ferritin and FerroOrange in HSCs in the BM of *Igf1*^*f/f*^ and *Igf1*^*ΔMK*^ mice with or without IGF1 supplementation at 3 dpi (*n* = 6). Data represent mean ± SD. **P < 0.01. One-way ANOVA

The redox-active ferrous iron (Fe^2+^) positions centrally in ferroptosis. The Fe^2+^ pool of a cell is primarily determined by iron uptake and Fe^2+^ release from ferritin protein by ferritinophagy [44]. Based on the transcriptomic analysis, we noticed that autophagy was robustly inhibited in HSCs of IGF1-treated mice (Figs. 5A and 6A), in line with the hyperactivation of mTOR, which is a well-known negative regulator of autophagy [5]. Utilizing GFP-LC3 reporter mice, we confirmed significantly inhibited autophagy in HSCs of IGF1- versus vehicle-treated mice (Fig. 6B). Concomitantly, the expression of ferritin protein was remarkably increased and the Fe^2+^ pool was remarkably reduced in HSCs of IGF1- versus vehicle-treated mice (Fig. 6C and D), while the total iron pool was comparable between IGF1- and vehicle-treated mice (Supplementary Fig. 6A), indicating a state of ferritinophagy inhibition. However, inhibition of mTOR by RAPA nearly abrogated the ferritinophagy inhibition in HSCs of IGF1-treated mice (Fig. 6B-D). As a result, the ferroptosis resistance of HSCs in IGF1-treated mice was abrogated by RAPA administration (Fig. 6E and F). In addition, although ferritinophagy in HSCs of *Igf1*^*ΔMK*^ and *Mpl*^*hlb219*^ mice was negligibly affected at homeostasis compared with that in their control counterparts (Fig. 6G, Supplementary Fig. 6B), it was remarkably increased post IR and could be reversed by IGF1 supplementation (Fig. 6H, Supplementary Fig. 6C). These results indicate that megakaryocytic IGF1 inhibits ferritinophagy via mTOR to restrict HSC ferroptosis after radiation injury.

### Punctual IGF1 administration effectively mitigates myelosuppression induced by radiation injury

**Fig. 7:**
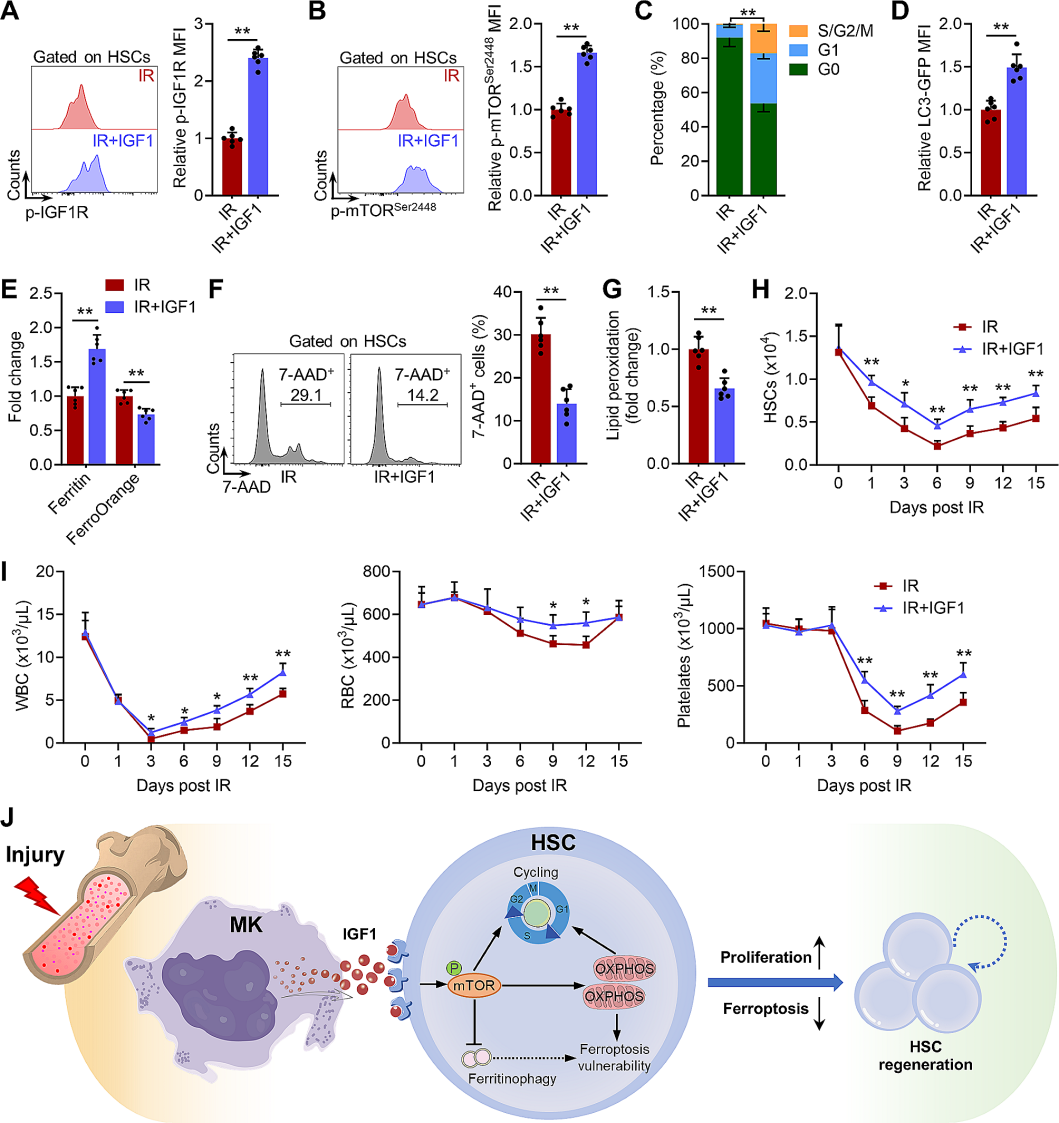
Punctual IGF1 administration effectively mitigates myelosuppression induced by radiation injury. (**A** and **B**) Flow cytometric analysis of expression of p-IGF1R and p-mTOR in HSCs in the BM of mice with or without IGF1 supplementation at 1dpi (*n* = 6). (**C**) Flow cytometric analysis of cell cycle of HSCs in the BM of mice with or without IGF1 supplementation at 1dpi (*n* = 6). (**D**) Flow cytometric analysis of GFP-LC3 expression in HSCs in the BM of GFP-LC3 mice with or without IGF1 supplementation at 1 dpi (*n* = 6). (**E**) Flow cytometric analysis of Ferritin and FerroOrange of HSCs in the BM of mice with or without IGF1 supplementation at 1 dpi (*n* = 6). (**F** and **G**) Flow cytometric analysis cell death and lipid peroxidation of HSCs in the BM of mice with or without IGF1 supplementation at 1dpi (*n* = 6). (**H**) The number of HSCs in the BM of mice with or without IGF1 supplementation at indicated time post IR (*n* = 6). (**I**) WBC, RBC, and platelet counts in the PB of mice with or without IGF1 supplementation at indicated time post IR (*n* = 6). (**J**) Schematic illustration of the protective role of megakaryocytic IGF1 in safeguarding HSC regeneration. Data represent mean ± SD. *P < 0.05, **P < 0.01. Two-tailed unpaired student’s *t*-test

As we previously reported, HSC ferroptosis peaks at 1 dpi [5]. The lag of megakaryocytic IGF1 hypersecretion may underlie the pathogenesis of IR-induced myelosuppression. We hypothesized that artificial elevation of IGF1 ahead of ferroptosis peak may effectively mitigate IR-induced myelosuppression. Then, we administered mice with a single dose of IGF1 immediately post IR. At 1 dpi, significantly increased activation of IGF1 signaling was detected in HSCs of IGF1-treated mice (Fig. 7A and B), accompanied by increased proliferation (Fig. 7C) and attenuated ferritinophagy (Fig. 7D and E) of HSCs. HSC ferroptosis at 1 dpi was significantly alleviated by IGF1 administration (Fig. 7F and G), resulting in improved regeneration of HSCs (Fig. 7H). Eventually, myelosuppression induced by IR was remarkably relieved by IGF1 administration (Fig. 7I). Therefore, unmasking megakaryocytic IGF1 hypersecretion dynamics provides a foundation for rational use of IGF1 to treat myelosuppression.

## Discussion

Despite substantial efforts to decipher the homeostatic regulatory network within the HSC niche, the reorganization and supportive role of HSC niche after injury remain poorly understood. Here we provide evidence that links the adaptive response of niche MKs to HSC regeneration using a mouse model of IR-induced myelosuppressive injury (Fig. 7J).

MKs are a population of HSC progeny that is quite distinct from the others due to their intimate interplay with HSCs [34]. Decades ago, MKs have been found to be resistant to even lethal cytotoxicity [12–14], while the underlying pathophysiological significance is unclear. Previously, we have shown that reprogramming of the apoptosis pathway during MK development contributes to the cytotoxicity-resistance of MKs. Meanwhile, in response to IR, MKs undergo extensive cellular and molecular remodeling, albeit they survive [15]. In the present study, we further reveal that the interplay between MKs and HSCs in the context of myelosuppressive injury is quite distinct from what is known at homeostasis. After myelosuppressive injury, MKs become a predominant component of HSC niche and elicit an adaptive response that is characterized by predominant and reversible IGF1 hypersecretion, which is necessary for optimal HSC regeneration. However, the secretion of TGF-β or CXCL4, which are predominantly sourced from MKs in HSC niche at homeostasis and are negative regulators of HSC activation [18, 20], is unchanged in MKs after myelosuppressive injury (Fig. 1H). Given the role of IGF1 in promoting HSC activation and regeneration as revealed in this study, from a teleological perspective, IGF1 hypersecretion by cytotoxicity-resistant MKs may be considered as a survival mechanism to preserve HSCs following cytotoxicity caused by IR, and perhaps by any form of myelosuppressive injury such as chemotherapy [18], while the mechanisms by which MKs distinctively reprogram their secretory profile in response to myelosuppressive injury warrants further investigation.

Systemic IGF1 is primarily sourced from the liver [45]. The exclusive elevation of IGF1 levels in the BM rather than in the circulation indicates that megakaryocytic IGF1 hypersecretion is governed by local instead of systemic factors post IR. Recently, STAT3, which is a well-known downstream effector of a variety of inflammatory cytokines and growth factors, is identified as a major positive regulator of *Igf1* transcription [41]. As known, the extensive BM destruction by myelosuppressive injury always invokes local inflammation, whose initiation and resolution respectively parallel HSC injury and regeneration [46]. Thus, the self-limited local inflammation potentially elicited by MK autocrine as we reported before [15] may dictate megakaryocytic IGF1 hypersecretion after injury. Notably, megakaryocytic IGF1 is dispensable for homeostatic HSC maintenance, hinting that systemic IGF1 or local IGF1 sourced from mesenchymal cells [37, 47, 48] is sufficient to maintain HSCs at homeostasis.

Although systemic IGF1 has been well recognized as a detrimental factor for mammal longevity, increasing studies have recently redefined local IGF1 as a supportive niche factor for self-renewal expansion of stem cells, including myogenic stem cell [39], embryonic stem cell [40], intestinal stem cells [41], and biliary epithelial cell [42]. However, the action of IGF1 on HSC maintenance is quite opposite as far as we know, as chronic IGF1 signaling activation has been demonstrated to exhaust HSCs [27, 38], while transient IGF1 stimulation can promote self-renewal and expansion of HSCs [22, 47]. In this study, we unravel that, in keeping with chronic IGF1 signaling activation, transient IGF1 stimulation activates mTOR to trigger HSC proliferation and mitochondrial oxidative metabolism, which fuels proliferation through supplying metabolic intermediates and energy [8, 9]. Notably, augmented mitochondrial oxidative metabolism usually provokes oxidative stress and subsequently enhances ferroptosis vulnerability of a cell [44]. Ingeniously, mTOR-mediated concomitant ferritinophagy inhibition restrains the availability of Fe^2+^ to diminish the overall ferroptosis vulnerability of HSCs. As a result, the delicate coordination between proliferation, mitochondrial oxidative metabolism and ferroptosis collectively ensures self-renewal and functional expansion of HSCs in response to transient IGF1 stimulation. As known, persistent oxidative stress as seen in chronic IGF1 signaling activation can induce ferritinophagy through alternative mechanisms [49], which will offset mTOR-mediated ferritinophagy inhibition. Therefore, ferroptosis vulnerability may represent as a key node to reconcile the contradiction between transient and chronic IGF1 signaling activation.

Generally, it is thought that in response to IR HSCs will immediately undergo apoptosis, which always peaks hours post IR, due to DNA damages induced by free radicals [7]. However, free radicals produced by ionization always are quenched within milliseconds, while HSC demise persists days post IR, implying the involvement of different death modes [50]. Indeed, we have recently reported that ferroptosis is a part of IR-induced HSC death [5]. Mechanistically, IR-induced redox imbalance including increased Fe^2+^ pool, decreased GSH/GSSG ratio, and ACSL4 upregulation may lead to lipid peroxidation and ferroptosis of HSCs [5, 51]. In addition, the inherent natures of HSCs such as glycolysis reliance and low protein synthesis rate actually make HSCs selectively vulnerable to ferroptosis [52]. In response to acute myelosuppressive injury, HSCs may be unable to timely remodel their nature. Especially, the metabolic rewiring to mitochondrial oxidative metabolism will further increase the ferroptosis vulnerability of HSCs [44, 53]. Besides, the profound intracellular damages induced by IR also provoke autophagy [54], which increases the ferroptosis vulnerability of HSCs as well. Consequently, although HSCs surviving IR-induced immediate apoptosis were dramatically proliferated from 1 dpi, they persistently suffer from ferroptosis, which continuously depletes HSCs by 6 dpi [5].

Fortunately, megakaryocytic IGF1 hypersecretion serves as a rheostat for HSC ferroptosis through inhibiting ferritinophagy. Thus, megakaryocytic IGF1 coordinates activation and ferroptosis vulnerability to ensure optimal HSC regeneration after myelosuppressive injury. Analogously, some cancers are also vulnerable to ferroptosis [53] and emerging studies have identified local IGF1 as a supportive factor for the maintenance of cancer stem cells [55, 56]. It is tempting to speculate that local IGF1 secretion may also serve as a ferroptosis defense mechanism for cancer stem cells. Of note, IGF1 signaling has been also demonstrated as a negative regulator of apoptosis [57]. Although ferritinophagy that can be inhibited by IGF1 signaling is specific for the process of ferroptotic cell death, given the complicated intersections among the diverse kinds of cell death including apoptosis and ferroptosis [58], further studies are still warranted to dissect the complexity of IR-induced HSC death and their regulation by IGF1 signaling.

Mammals usually recover rapidly from acute myelosuppression with hematopoietic growth factor treatment, the principle therapeutic strategy for myelosuppression, but at the expense of HSC exhaustion [59]. Our study suggests a therapeutic superiority of IGF1 against myelosuppression, as it simultaneously promotes HSC regeneration and hematopoietic recovery. Actually, IGF1 has been approved for use in human by the U.S. Food and Drug Administration decades ago. Meanwhile, the effectiveness of IGF1 in mitigating lethal myelosuppression has also been evaluated decades ago [60, 61], while the therapeutic time window and regimen of IGF1 remain uncertain. In this study, based on the temporal dynamics of HSC regeneration and megakaryocytic IGF1 hypersecretion, we propose that a single dose of IGF1 administration immediately after myelosuppressive injury is efficient in exerting therapeutic effect. In addition, HSC ferroptosis is also implicated in other human diseases characterized by HSC loss such as Fanconi anemia and aplastic anemia [52], highlighting a broader therapeutic potential of IGF1 in a number of BM failure syndromes.

## Conclusions

In summary, this work identifies megakaryocytic IGF1 as a critical niche signal safeguarding HSC regeneration after radiation injury. These findings not only provide novel insights into the MK-HSC interaction in the context of myelosuppressive injury, but also extend our understanding of the action of local IGF1 in stem cell maintenance, which have broad and valuable implications for the management of radiation injury, BM failure syndrome and cancer.

### Electronic supplementary material

Below is the link to the electronic supplementary material.

Supplementary Material 1

## Data Availability

RNA-seq data generated during this study have been deposited at GEO (Accession numbers: GSE222512, GSE244971, GSE229985) and are publicly available as of the date of publication.

## Abbreviations

BM: Bone marrow
HSC: Hematopoietic stem cell
BMC: BM cell
IR: Ionizing radiation
OXPHOS: Oxidative phosphorylation
dpi: Day post IR
MK: Megakaryocyte
IGF1: Insulin-like growth factor 1
MkP: MK progenitor
nMK: Niche-supporting MK
iMK: Immune MK
pMK: Platelet-producing MK
RAPA: Rapamycin
Fe^2+^: Ferrous iron
