# Supplementary material for: Megakaryocytic IGF1 coordinates activation and ferroptosis to safeguard hematopoietic stem cell regeneration after radiation injury
Source: Cell Commun Signal. 2024 May 27;22:292. doi: 10.1186/s12964-024-01651-5 (PMC11129484; doi:10.1186/s12964-024-01651-5)
Supplement: Supplementary file 1 — Supplementary Material 1 [file 12964_2024_1651_MOESM1_ESM.docx]

Supplementary material for

Megakaryocytic IGF1 coordinates activation and ferroptosis to safeguard hematopoietic stem cell regeneration after radiation injury

Liao et al.

Correspondence to: changhongdu@tmmu.edu.cn

**This PDF file includes:**

Supplementary Tables

Supplementary Figures

**Supplementary Tables**

**Supplementary Table 1. Antibodies used for flow cytometry.**

| **Antibody** | **Origin** | **Catalog number** |
| --- | --- | --- |
| FITC Mouse Hematopoietic Lineage Cocktail | eBioscience | Cat# 22-7770-82 |
| Biotin Mouse Hematopoietic Lineage Cocktail | eBioscience | Cat# 88-7774-75 |
| PerCP-Cyanine5.5 Anti-Mouse Ly-6A/E (Sca-1) | eBioscience | Cat# 45-5981-82 |
| PE anti-mouse Ly-6A/E (Sca-1); E13-161.7 | Biolegend | Cat#122508 |
| APC-eFluor 780 Anti-Mouse CD117 (c-Kit) | eBioscience | Cat# 47-1171-82 |
| PE-Cyanine7 Anti-Mouse CD117 (c-Kit) | eBioscience | Cat# 25-1171-82 |
| APC Anti-Mouse CD117 (c-Kit) | eBioscience | Cat# 17-1171-83 |
| PE Anti-Mouse CD135 (Flt3) | eBioscience | Cat# 12-1351-83 |
| APC Anti-Mouse CD135 (Flt3) | eBioscience | Cat# 17-1351-82 |
| eFluor 660 Anti-Mouse CD34 | eBioscience | Cat# 50-0341-82 |
| FITC Anti-Mouse CD34 | eBioscience | Cat# 11-0341-85 |
| FITC Anti-Mouse CD41 | eBioscience | Cat# 11-0411-85 |
| APC Anti-Mouse CD41 | eBioscience | Cat# 17-0411-80 |
| Biotin Anti-Mouse CD41 | eBioscience | Cat# 13-0411-82 |
| PE Anti-Mouse CD42d | eBioscience | Cat# 12-0421-82 |
| eFluor 450 Anti-Mouse CD45 | eBioscience | Cat# 48-0451-82 |
| BV605 Anti-Mouse CD45.1 | eBioscience | Cat# 12-0453-82 |
| FITC Anti-Mouse CD45.2 | eBioscience | Cat# 11-0454-82 |
| APC-eFluor 780 Anti-human/mouse CD45R (B220) | eBioscience | Cat# 47-0452-82 |
| PE-Cyanine7 Anti-Mouse CD3e | eBioscience | Cat# 25-0031-82 |
| PE Anti-Mouse Ly-6G (Gr-1) | eBioscience | Cat# 12-5931-82 |
| APC Anti-Mouse CD11b (Mac-1) | eBioscience | Cat# 17-0112-82 |
| FITC Anti-Mouse CD3e | eBioscience | Cat# 11-0031-82 |
| APC Anti-Mouse CD3e | Biolegend | Cat#100312 |
| Brilliant Violet 510™ Streptavidin | Biolegend | Cat# 405234 |
| eFluor™ 660 Anti-Phospho-mTOR (Ser2448) Monoclonal Antibody (MRRBY) | eBioscience | Cat#50-9718-42 |
| eFluor 450 Anti-Human/Mouse phospho-AKT (S473) | eBioscience | Cat# 48-9715-42 |
| FITC Anti-Mouse/Human IGF1 | Thermo Fisher Scientific | Cat# MA5-46576 |
| Alexa Fluor 647 Anti- phospho-IGF1R (Tyr1131) | BD Phosflow | Cat# 558588 |
| Anti-Ferritin antibody [EPR3004Y] | Abcam | Cat# ab75973 |
| Goat anti- Rabbit IgG(H+L) Cross-Adsorbed Secondary Antibody, Alexa Fluor 488 | Thermo Fisher Scientific | Cat# A-11008 |
| Goat anti-Rabbit IgG (H+L) Highly Cross-Adsorbed Secondary Antibody, Alexa Fluor 594 | Thermo Fisher Scientific | Cat# A-11037 |
| PE Anti-Mouse/Rat Ki-67 | eBioscience | Cat# 12-5698-82 |
| eFluor 450 Anti-Mouse CD45 | eBioscience | Cat# 48-0451-82 |
| PE-Cyanine7 Anti-Mouse TER-119 | eBioscience | Cat# 25-5921-82 |
| PerCP-Cyanine5.5 Anti-Mouse CD11c | eBioscience | Cat# 45-0114-82 |
| PE-Cyanine7 Anti-Mouse CD31 | eBioscience | Cat# 25-0311-82 |
| PE Anti-Mouse CD51 | eBioscience | Cat# 12-0512-82 |
| Anti-Mouse MYLK4 | Abmart | Cat# PK43039 |
| Anti-Mouse LSP1 | Abmart | Cat# T55653 |
| Anti-Mouse ARNTL | Abmart | Cat# MG791277 |

**Supplementary Table 2.** **Primers for mRNA expression analysis.**

| **Gene** | **Forward primer (5’ –3’)** | **Reverse primer (5’ –3’)** |
| --- | --- | --- |
| *Igf1* | CCGAGGGGCTTTTACTTCAAC | CAGTCTCCTCAGATCACAGCT |
| *Hprt* | TCAGTCAACGGGGGACATAAA | GGGGCTGTACTGCTTAACCAG |

**Supplementary Figures**


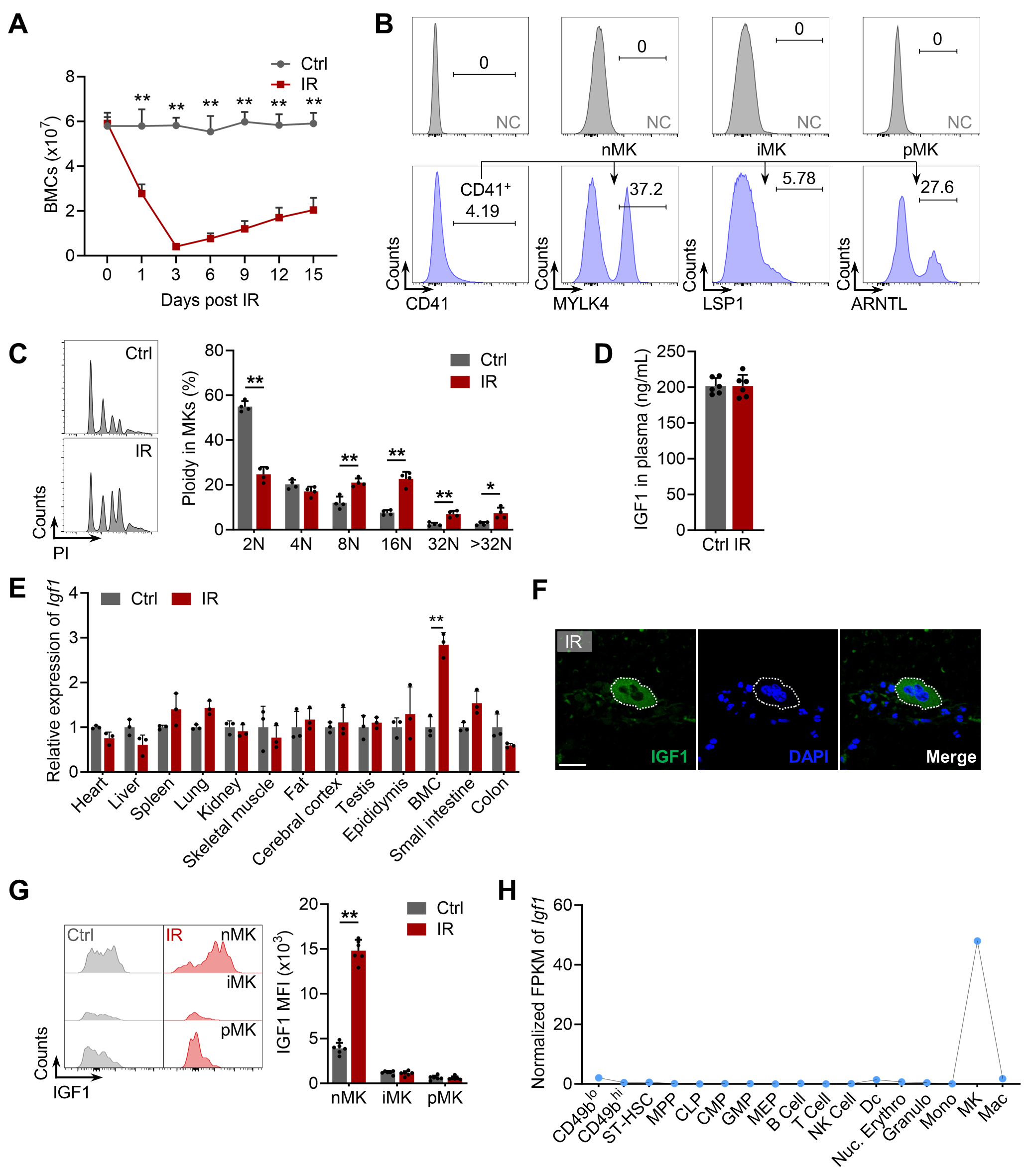


**Supplementary Fig. 1 Alteration of BMCs and IGF1 contents under homeostasis and stresses. A**, The number of BMCs at indicated time post IR (*n* = 6). **B**, Representative flow cytometric analysis of MK subpopulations. **C**, Flow cytometric analysis of MK ploidy distribution at 3 dpi (*n* = 4). **D**, Relative IGF1 levels in the plasma of mice at 3 dpi (*n* = 6). **E**, Relative *Igf1* expression in different tissues at 3 dpi (*n* = 6). **F**, Immunofluorescence analysis of IGF1 production by BMCs of IR mice at 3 dpi. The dashed line outlines MK. Scale bar, 20 μm. **G**, Flow cytometric analysis of IGF1 expression in MK subpopulations at 3 dpi. **H**, Normalized FPKM of *Igf1* in different hematopoietic cell types in the mouse BM. (CD49b^lo^, CD49b^lo^ HSC; CD49b^hi^, CD49b^hi^ HSC; ST-HSC, short-term HSC; MPP, multipotent progenitor; CLP, common lymphoid progenitor; CMP, common myeloid progenitor; GMP, granulocyte-macrophage progenitor; MEP, megakaryocyte-erythroid progenitor; Nuc. Erythro, nucleated erythrocyte; Granulo, granulocyte). Data represent mean ± SD. ***P* < 0.01. Two-tailed unpaired student’s *t*-test.


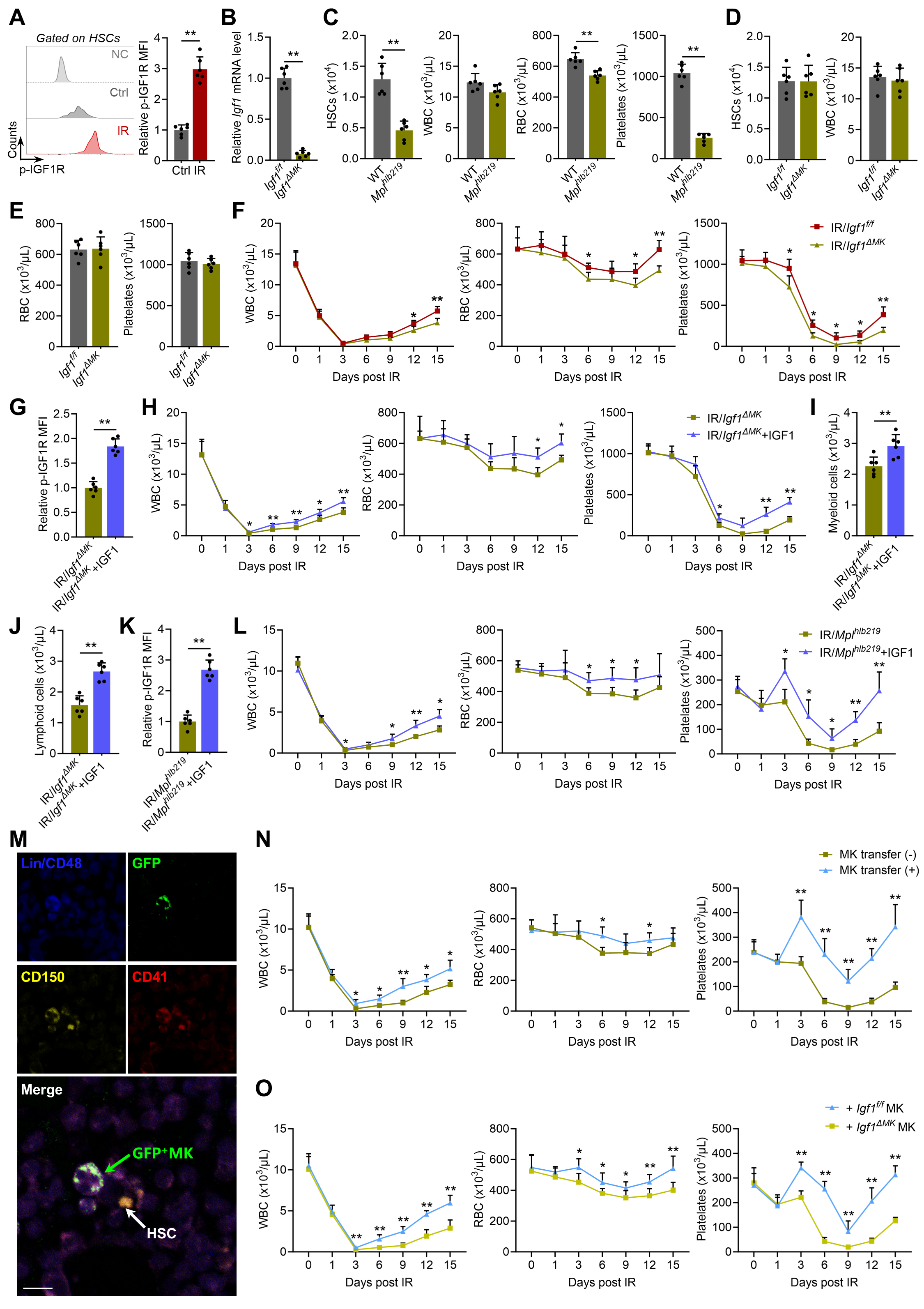


**Supplementary Fig. 2 Megakaryocytic IGF1 secretion favors hematopoiesis regeneration after radiation injury. A**, Flow cytometric analysis of p-IGF1R expression in HSCs in the BM of mice at 3 dpi (n = 6, NC: negative control). **B**, Relative *Igf1* expression of MKs in the BM from *Igf1^fl/fl^* and *Igf1^ΔMK^* mice (*n* = 6). **C**-**E**, The number of HSCs in the BM and white blood cell (WBC), red blood cell (RBC), and platelet counts in the PB of *Mpl^hlb219^* and *Igf1^ΔMK^* mice (*n* = 6). **F**, WBC, RBC, and platelet counts in the PB of *Igf1^fl/fl^* and *Igf1^ΔMK^* mice at indicated time post IR (*n* = 6). **G**, Flow cytometric analysis of p-IGF1R expression in HSCs in the BM of *Igf1^ΔMK^* mice with or without IGF1 supplementation at 3 dpi (*n* = 6). **H**, WBC, RBC, and platelet counts in the PB of *Igf1^ΔMK^* mice with or without IGF1 supplementation at indicated time post IR (*n* = 6). **I** and **J**, Myeloid cells and lymphoid cells counts in the PB of *Igf1^ΔMK^* mice with or without IGF1 supplementation at indicated time post IR (*n* = 6). **K**, Flow cytometric analysis of p-IGF1R expression in HSCs in the BM of *Mpl^hlb219^* mice with or without IGF1 supplementation at 3 dpi (*n* = 6). **L**, WBC, RBC, and platelet counts in the PB of *Mpl^hlb219^* mice with or without IGF1 supplementation at indicated time post IR (*n* = 6). **M**, Immunostaining analysis of location of transferred GFP^+^ MK and HSC in the BM of IR-exposed *Mpl^hlb219^* mice with adoptive MK transfer. Scale bar, 20 μm. The green arrow indicates GFP^+^ MK. The white arrow indicates HSC. **N**, WBC, RBC, and platelet counts in the PB of *Mpl^hlb219^* mice with or without MK transfer at indicated time post IR (*n* = 6). **O**, WBC, RBC, and platelet counts in the PB of *Mpl^hlb219^* mice with *Igf1^fl/fl^* or *Igf1^ΔMK^* mice-derived MK transfer at indicated time post IR (*n* = 6). Data represent mean ± SD. **P* < 0.05, ***P* < 0.01. Two-tailed unpaired student’s *t*-test.


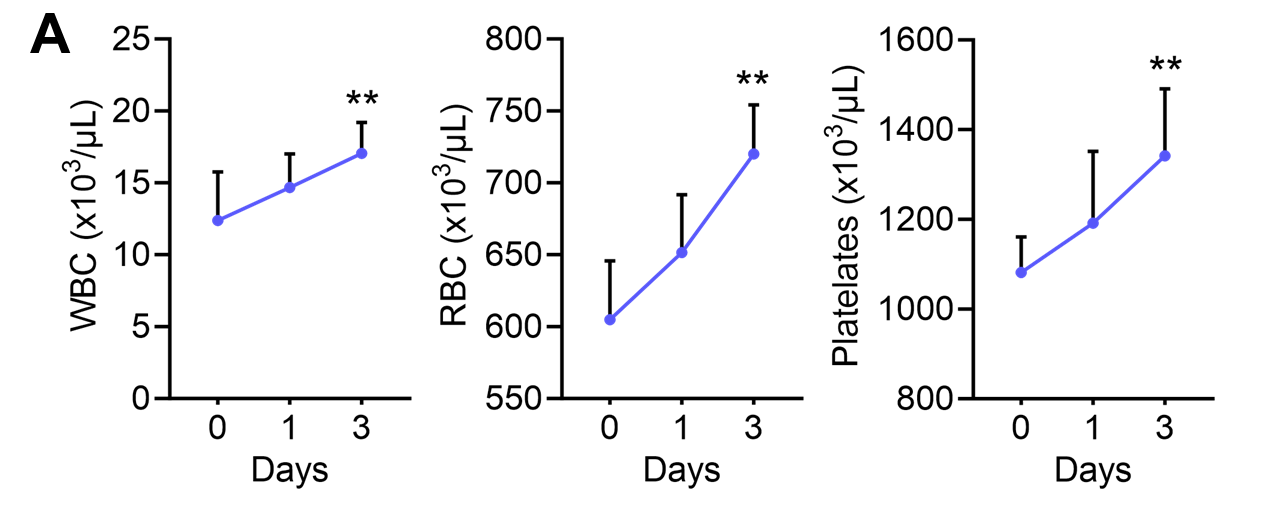


**Supplementary Fig. 3 IGF1 promotes homeostatic hematopoiesis. A**, WBC, RBC, and platelet counts in the PB of mice at indicated time post IGF1 administration (*n* = 6). Data represent mean ± SD. ***P* < 0.01. One-way ANOVA, compared with day 0 post IGF1 administration.


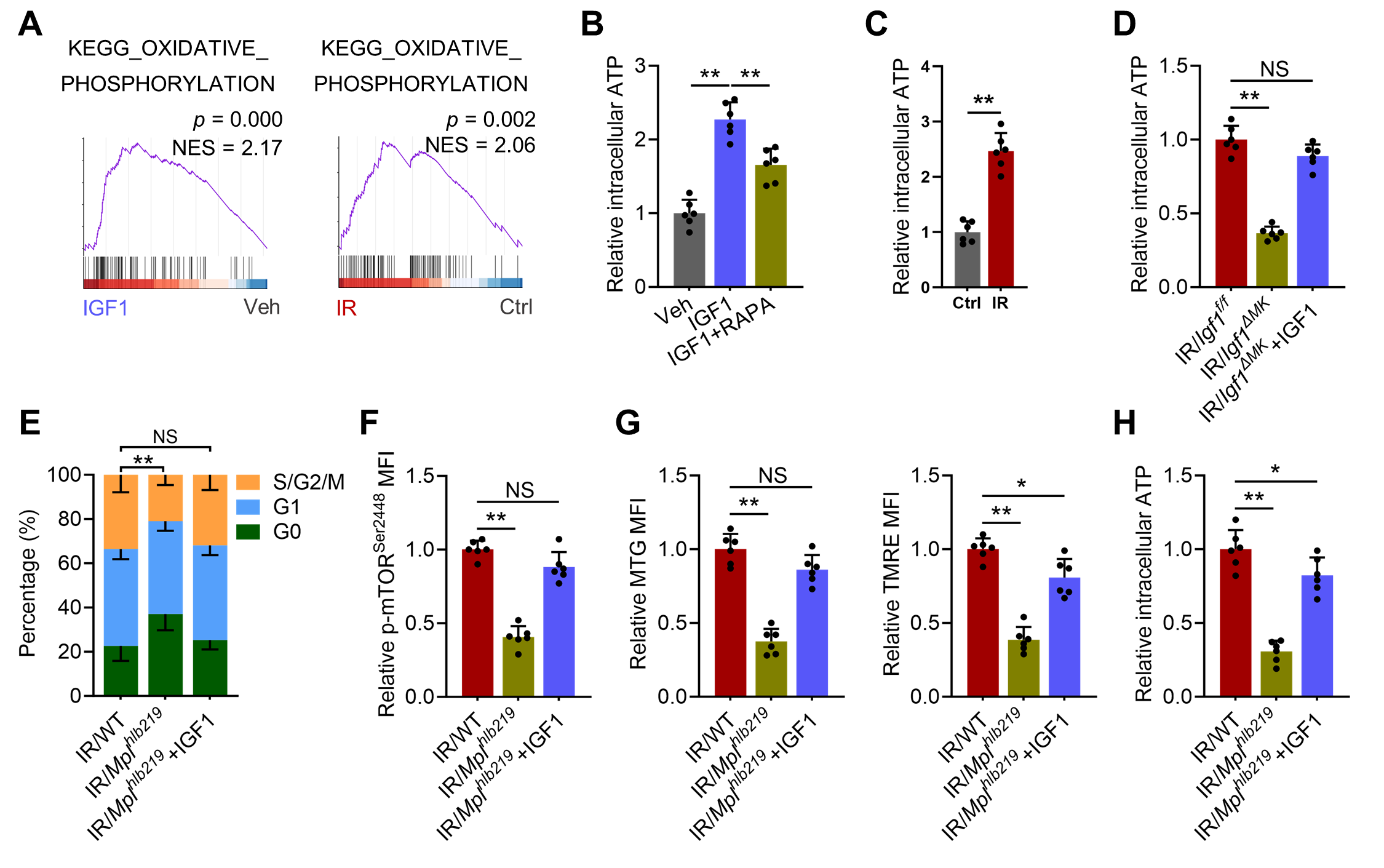


**Supplementary Fig. 4 Megakaryocytic IGF1 secretion promotes activation of HSCs. A**, GSEA of oxidative phosphorylation gene set in BM HSCs of mice at day 1 post IGF1 administration (IGF1 vs vehicle) and at 3 dpi (IR vs control). **B**, Intracellular ATP assay of HSCs in the BM of mice at 1 day post IGF1/RAPA administration (*n* = 6). **C**, Intracellular ATP assay of HSCs in the BM of mice at 3 dpi (*n* = 6). **D**, Intracellular ATP assay of HSCs in the BM of *Igf1^f/f^* and *Igf1^ΔMK^* mice with or without IGF1 supplementation at 3 dpi (*n* = 6). **E-G**, Flow cytometric analysis of cell cycle, p-mTOR expression, MTG and TMRE in HSCs in the BM of WT and *Mpl^hlb219^* mice with or without IGF1 supplementation at 3 dpi (*n* = 6). **H**, Intracellular ATP assay of HSCs in the BM of WT and *Mpl^hlb219^* mice with or without IGF1 supplementation at 3 dpi (*n* = 6). Data represent mean ± SD. **P* < 0.05, ***P* < 0.01, NS: no significance. One-way ANOVA unless stated otherwise. Two-tailed unpaired student’s *t*-test was used for calculating *P* values in C.


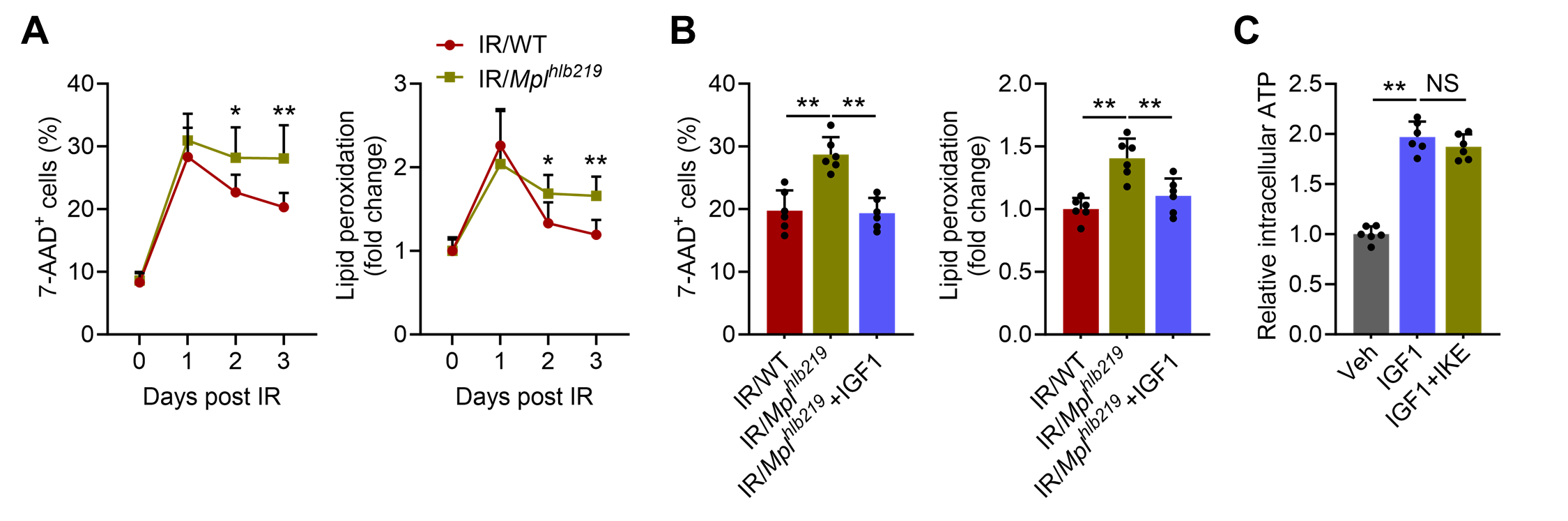


**Supplementary Fig. 5 Megakaryocytic IGF1 secretion diminishes activation-associated ferroptosis vulnerability of HSCs. A**, Flow cytometric analysis of cell death and lipid peroxidation of HSCs in the BM of WT and *Mpl^hlb219^* mice at indicated time post IR (*n* = 6). **B**, Flow cytometric analysis of cell death and lipid peroxidation in HSCs in the BM of WT and *Mpl^hlb219^* mice with or without IGF1 supplementation at 3 dpi (*n* = 6). **C**, Intracellular ATP assay of HSCs in the BM of mice at 1 day post IGF1/IKE administration (*n* = 6). Data represent mean ± SD. **P* < 0.05, ***P* < 0.01, NS: no significance. One-way ANOVA unless stated otherwise. Two-tailed unpaired student’s *t*-test was used for calculating *P* values in A.


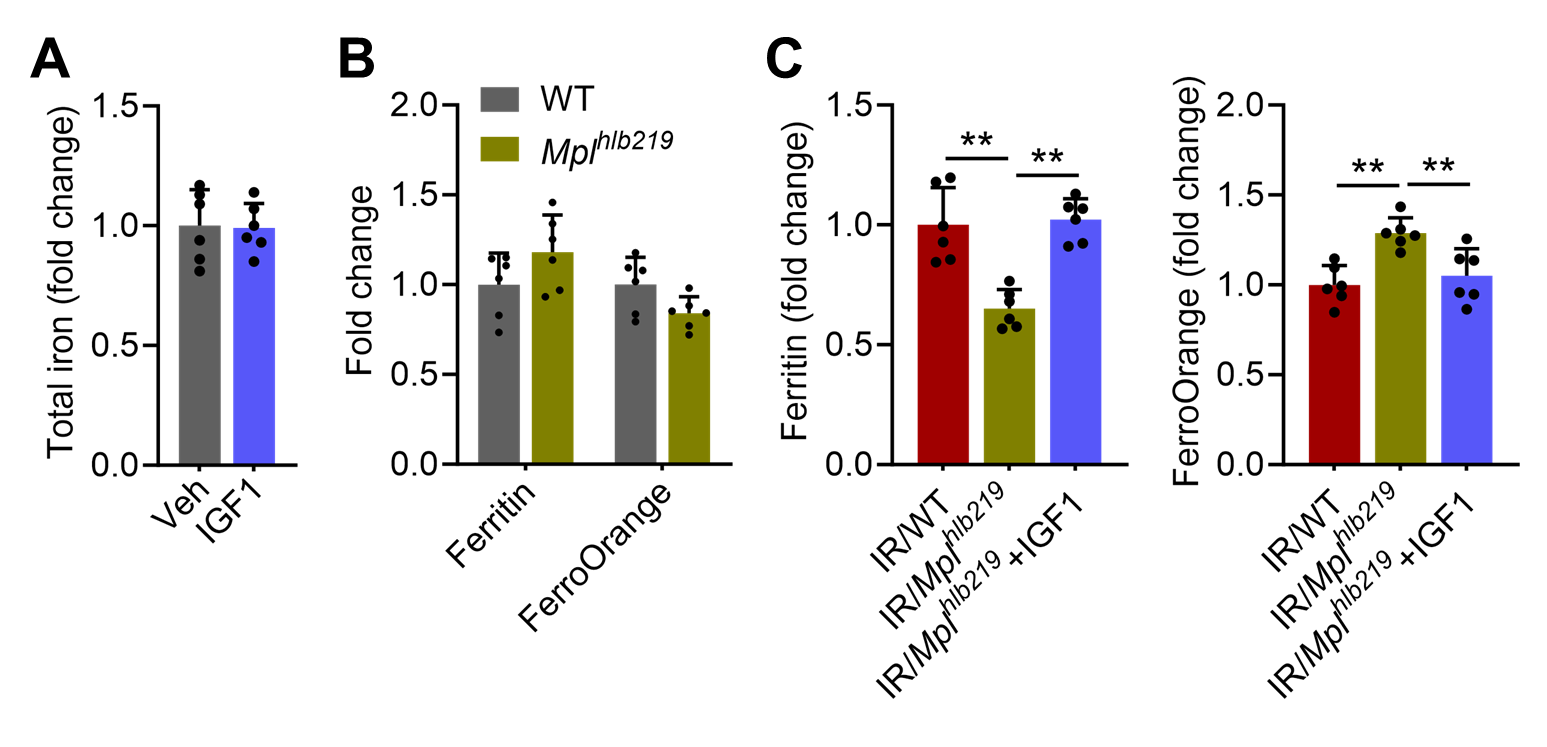


**Supplementary Fig. 6 IGF1 inhibits ferritinophagy in HSCs via mTOR. A**, Relative total iron contents in BM HSCs of mice at 1 day post IGF1 supplementation (*n* = 6). **B**, Flow cytometric analysis of Ferritin and FerroOrange in HSCs in the BM of WT and *Mpl^hlb219^* mice (*n* = 6). **C**, Flow cytometric analysis of Ferritin and FerroOrange in HSCs in the BM of WT and *Mpl^hlb219^* mice with or without IGF1 supplementation at 3 dpi (*n* = 6). Data represent mean ± SD. ***P* < 0.01. One-way ANOVA.
